# Fault Recovery-Oriented Dynamic Service Migration Deployment of UGV Formations

**DOI:** 10.3390/s26216828

**Published:** 2026-10-29

**Authors:** Han Panpan, Ding Dali, Mao Yong, Liu Yidong, Zhang Qi

**Affiliations:** 1 Aviation Engineering School, Air Force Engineering University, Xi’an 710038, Chinazhangqi19910527@163.com (Z.Q.); 2 Jiangnan Institute of Electromechanical Design, the 10th Research Institute, China Aerospace Science and Industry Corporation, Guiyang 550009, China

**Keywords:** UGV formation, dynamic service migration, whale optimization algorithm (WOA), reinforcement learning, fault recovery

## Abstract

**Highlights:**

**What are the main findings?**

- A fault-severity-aware dual-strategy framework is developed for UGV formations, switching between WOA-based intra-vehicle priority migration and SMAG-DQN-based inter-vehicle load-balancing migration according to residual processor capacity, failed-service criticality, and mission phase.
- WOA achieves the lowest migration delay and energy consumption under mild intra-vehicle faults, while SMAG-DQN delivers a low-latency multi-objective trade-off for inter-vehicle migration by integrating spectral-clustering-based migration atomic groups with deep reinforcement learning.

**What are the implications of the main findings?**

- The proposed framework enables on-demand adaptation between migration overhead and load-balancing performance, supporting rapid fault recovery and service continuity in cooperative UGV formations under random processor faults.
- The analytical transmission-delay operator and clustering-reduced decision space help overcome the precision limits of static deployment models and provide a pathway toward validation on real open-architecture UGV platforms.

**Abstract:**

To address the issue of vehicle functionality loss caused by processor failure in unmanned vehicle cooperative formation scenarios, this paper constructs a fault-severity-aware and scenario-adaptive migration strategy that dynamically matches the actual severity of each fault event to accommodate service migration requirements across diverse scenarios. A multi-objective evaluation model is established, incorporating migration energy cost, load balancing rate, and service migration time, to clearly define the optimization objectives for service migration strategies. We propose Whale Optimization Algorithm (WOA) to solve the multi-objective optimization problem for intra-vehicle migration and to resolve the inherent difficulty of simultaneously achieving scheduling effectiveness, rapid responsiveness, and adaptability to dynamic environments in inter-vehicle fault-induced service migration. We further develop an adaptive DQN-based dynamic service migration method with spectral clustering-based migration atomic groups (SMAG-DQN). SMAG-DQN introduces a spectral clustering-based migration atomic group constraint mechanism, integrating spectral clustering with deep reinforcement learning to construct a decision-making framework. By leveraging clustering to reduce the decision space, it effectively compresses computation time while maintaining scheduling performance. The results demonstrate that the proposed dynamic service migration strategy for UGV formations achieves significant advantages in balancing multi-dimensional performance metrics under dynamic conditions. Specifically, the WOA-based strategy attains a migration delay of 118 ms and energy cost of 8 J with 100% intra-vehicle coverage, while the SMAG-DQN-based strategy completes each decision in 0.55 s, compresses the decision chain from 78 to 15–20 steps, and achieves a delay coefficient of variation of 5.7%, demonstrating strong decision stability and effectively supporting fault recovery in cooperative UGV formation operations.

## 1. Introduction

Cooperative combat of UGV formations has become a critical paradigm in Internet of Vehicles, smart cities, and intelligent battlefield operations. The vehicles within the formation operate in coordination through onboard processors, carrying out hundreds of command, detection, navigation and autonomous maneuvering services, forming a mobile distributed system. Typically, the mission of a UGV formation is defined by the decision-making layer, which assigns task profiles to each vehicle, with different vehicles equipped with distinct payload modules according to their roles.

In recent years, the widespread adoption of open architectures and service-oriented paradigms in vehicle-mounted and avionics systems [1] has spurred research into service-oriented flexible architectures for unmanned ground vehicles. By partitioning the functional units of each unmanned combat vehicle into manageable resources, this approach effectively encapsulates and integrates the distributed applications and resources within the system, thereby forming a unified resource pool. Depending on the specific combat mission, services can be rapidly composed to build complex cross-organizational applications [2], ultimately enabling flexible system integration.

Among these, the static service deployment strategy for UGVs aims to achieve optimal matching between services, such as perception, localization, planning, and control, and onboard processor resources. Current research has conducted mapping planning for hundreds of encapsulated services to their hosting servers [3]. However, in cooperative UGV formations, due to the influence of the surrounding environment and product reliability, individual UGVs or certain processors may fail. Once a processor on a UGV suddenly fails, the services originally deployed on it will completely lose their functionality. Moreover, since other services also depend on the outputs of the faulty processor, the entire vehicle faces a high risk of complete functional loss, which significantly degrades the formation’s effectiveness.

In existing cooperative UGV formations, once the command center detects a processor failure, it must immediately migrate the services originally hosted on the faulty processor to other healthy processors in real time; otherwise, the formation chain will directly break down and the mission will be interrupted. The essence of faulty service migration lies in rapidly reconfiguring the mapping between the failed services and the remaining healthy processors under the constraint of limited inter-vehicle communication bandwidth. This process demands a high degree of flexibility and autonomy, which is enabled by the service management layer and the cooperation layer built upon the open architecture of each individual vehicle.

To improve the fault tolerance and availability of service clusters in cooperative UGV formations, many researchers have adopted a certain proportion of backup images to ensure timely service migration [4]. When some processors fail, the objective of the service migration strategy is to minimize the communication power cost, service migration time, and processor load imbalance, subject to the processing capacity constraints of the onboard processors. In addition, considering that a UGV with a faulty processor is highly likely to suffer failures on other processors as well, the same-vehicle migration ratio should be kept as small as possible. These measures aim to ensure rapid recovery of faulty functions while maintaining balanced processor loads. Compared with static service deployment strategies, dynamic service migration for fault scenarios in UGV formation cooperation also necessitates lightweight and rapid decision-making capabilities.

Challenges of dynamic service migration for fault scenarios in UGV formation cooperation include the following:

Fault recovery relies on pre-deployed service image backups; services without a backup must transmit full images (tens of MB) over bandwidth-limited inter-vehicle links, incurring hundreds of milliseconds of transfer delay. The migration target is thus jointly constrained by topological proximity and image distribution.

Healthy processors already run existing loads, and collateral damage raises the probability of secondary failures on the same vehicle. Every feasible scheme must keep each processor within its residual capacity while minimizing the same-vehicle migration ratio.

Aggressive nearest-neighbor migration overloads the target and degrades load balance, whereas even distribution increases transmission distance, power, and inter-vehicle interaction frequency.

These challenges are not adequately addressed by existing methods, which typically lack fault-severity awareness and make decisions at the per-service granularity. To the best of our knowledge, no prior work simultaneously considers fault-severity adaptation, multi-objective balance, and group-granularity decisions for fault-induced service migration in UGV formations.

This paper makes the following contributions:

1. We establish a three-dimensional evaluation model (migration energy cost, load-balancing rate, and migration time) that guides adaptive selection between hot and cold migration.
2. We propose a fault-severity-aware dual-strategy method: WOA-based intra-vehicle migration for mild faults, and SMAG-DQN—spectral-clustering-derived migration atomic groups (MAGs) combined with deep reinforcement learning—for inter-vehicle load-balancing migration, raising the decision granularity from per-service to MAG level.
3. We benchmark the method against NSGA-II, a Gibbs-sampling/Lyapunov method, and per-service DQN, showing that SMAG-DQN compresses the decision chain from 78 to 15–20 steps with a 5.7% delay coefficient of variation.

The main novelty is a fault-severity-aware dual-strategy framework that switches between WOA-based intra-vehicle migration and SMAG-DQN-based inter-vehicle migration according to residual capacity, failed-service criticality, and mission phase. For mild faults in which the faulty vehicle retains sufficient residual capacity, a WOA-based intra-vehicle migration strategy is adopted. The framework further elevates the DQN decision granularity from per-service to spectral-clustering-derived migration atomic groups (MAGs) with cohesion/dispersion biases—a combination that has not been reported for UGV formation fault recovery.

## 2. Related Work

Dynamic service migration has broad application prospects in low-latency and highly dynamic scenarios such as the Internet of Vehicles, smart cities, and augmented reality. Current research has expanded from mathematical optimization and heuristic search to reinforcement learning and hybrid approaches.

In terms of mathematical optimization algorithms, Shangguang Wang et al. [4] formulated the problem with the objectives of minimizing user access latency and balancing server loads, and employed mixed-integer programming (MIP) to solve for the optimal placement scheme. Ao Zhou [5] addressed the joint optimization of service entity placement, user association, and resource allocation as a mixed-integer nonlinear programming problem, and proposed a two-layer iterative algorithm for its solution: the outer layer optimizes user association via Gibbs sampling, while the inner layer iteratively solves for transmission power allocation and service entity placement through convex optimization and Lagrangian duality, with an approximate optimal solution obtained via simulated annealing. ALBLA [6] focuses on dynamic load balancing in edge cloud environments, adopting a decentralized architecture and learning automata to achieve distributed adaptive scheduling. It maintains a list of available servers and a probability vector to allocate tasks between the edge and the cloud in real time, and updates the probabilities based on service time feedback, thereby effectively reducing latency, improving edge execution rates, and achieving load balancing.

In the field of heuristic search, Hui Ma et al. [7] addressed the multi-objective optimization problem of application deployment in multi-cloud environments by proposing an NSGA-II algorithm integrated with local search. They pursued dual optimization objectives of minimizing total response time and minimizing total deployment cost, and solved the problem using non-dominated sorting, semi-uniform crossover, bit-flip mutation, and dynamic probabilistic local search. ACO et al. [8] adopted ant colony optimization combined with heuristic algorithms for joint optimization, locating the nearest server via Dijkstra’s algorithm and assigning tasks according to the load-to-computing-capacity ratio, with scheduling prioritized by task completion time, thereby effectively balancing loads and reducing response time. Chen Ling’s team [9] employed GCN for network traffic prediction in edge server resource utilization calculation, and used a weighted objective function that normalizes Euclidean distance and base station traffic utilization as the optimization target, with particle swarm optimization applied to accomplish edge server deployment. Wei Huang et al. [10] addressed the joint placement problem of edge servers and UAVs under dynamically changing network environments and computational resource demands, and proposed a heterogeneous server dynamic deployment scheme. They combined particle swarm optimization with genetic algorithm (GAPSO) to search for the optimal deployment of edge servers and UAVs, and adopted the KM algorithm to compute the minimum migration energy cost between old and new server locations. Yan et al. [11] focused on low-earth-orbit (LEO) satellite networks in the Internet of Space Things (IOST). For static service deployment, they deployed a number of servers to minimize the weighted average latency across all snapshots over the full cycle. For the dynamic edge server deployment problem, they pre-installed servers on all satellites and controlled their on/off status to minimize the average response latency of the current snapshot, and proposed a genetic algorithm to find the optimal solution. However, the dynamic service migration approach that pre-installs servers on all satellites incurs severe resource waste.

Many researchers have constructed multi-layer policy architectures to improve the rationality and dynamic responsiveness of service deployment. Chenyang Wang et al. [12] built a UE-Edge-Cloud three-tier architecture, modeled the microservice deployment problem as a max-min optimization jointly considering cost and QoS, and proposed an attention-based microservice representation (AMR) to efficiently extract system features by filtering out redundant infrastructure information and retaining effective contextual information. They further designed an attention-improved soft actor-critic (ASAC) deep reinforcement learning algorithm to solve the problem. Liu et al. [13] formulated the joint optimization of static and dynamic edge server deployment, user access, and service cost in mobile edge computing as an optimization problem with latency constraints, and solved it using an improved snake optimization algorithm (ISO). The outer layer optimizes the number and positions of dynamic servers via PSO, bidirectional search, and an elite leadership strategy to meet latency requirements, while the inner layer minimizes server movement distance based on linear programming and the interior-point method, combined with a dynamic adjustment mechanism to achieve near-optimal deployment. Ali Asghari et al. [14] similarly adopted a hierarchical design philosophy, dividing the area into geographical partitions in advance, performing a coarse global search using WOA, followed by local fine-tuning with neural networks, and finally achieving global equilibrium through game theory.

In terms of reinforcement learning applications, the DRLO team [15] set the top-level objective of minimizing the maximum average access latency in dynamic MEC networks, constructed an MDP using DDPG reinforcement learning, designed a latency-centric reward function, and employed improved activation functions to enhance exploration capability in large action spaces, thereby achieving service deployment in dynamic environments. Shihua Li et al. [16] proposed an adaptive placement and dynamic optimization method based on deep Q-networks (SMAG-DQN), and designed a load-sensitive placement rule (LSD) to address the key issues of exponential complexity growth with the number of servers and poor adaptability. Anum Talpur [17] constructed a lightweight rapid dynamic service migration framework by eliminating future rewards and combining ILP cold-start with an active re-optimization triggering mechanism. Sonkoly et al. [18] reviewed placement strategies for edge computing.

AQEEL H. KAZMI et al. compared various algorithms—including genetic algorithm, simulated annealing, particle swarm optimization, ant colony optimization, artificial bee colony, and reinforcement learning—against a multi-objective weighted comprehensive optimization target for edge server configuration and user requests, and evaluated their decision-time performance. In terms of single-objective optimization, particle swarm optimization, simulated annealing, and reinforcement learning demonstrated superior performance [19]. Peng et al. [20] explored cost-aware service migration in vehicular edge networks.

Furthermore, Qiu et al. addressed the service provisioning problem of immersive virtual vehicles in edge-enabled vehicular metaverse (EVM) scenarios. For static service backup deployment, they deployed multiple groups of backup virtual vehicle services to ensure operational reliability, and proposed an integrated spot lighter framework that combines Transformer-based prediction, randomized rounding migration, and consumption-adaptive AOBI-guaranteed mechanisms to obtain suboptimal feasible solutions for dynamic vehicle service scheduling [21].

However, the above studies pay insufficient attention to the timeliness of dynamic service migration decisions under varying environments, and research specifically on dynamic service migration in UGV formations remains scarce, with most existing work concentrating on SOA architectures and related aspects. In edge computing scenarios with stringent rapid requirements, such as UGV operations, where a processor may fail at any time, the majority of reference solutions for edge services rely on heuristic or reinforcement learning algorithms. These approaches often suffer from imbalanced multi-objective optimization and yield unsatisfactory performance under strong timeliness constraints. Alternatively, lightweight algorithmic modifications that shorten simulation time typically adopt weighted comprehensive objectives as the optimization target, which cannot guarantee optimality across multiple objectives simultaneously. Existing service migration strategies lack diversity.

In contrast to the DQN/DRL-based methods reviewed above, which target generic edge or vehicular service placement and make decisions at the per-service granularity, the proposed SMAG-DQN elevates the decision granularity to spectral-clustering-derived migration atomic groups (MAGs) with cohesion and dispersion biases, and is embedded in a fault-severity-aware dual-strategy framework specifically designed for UGV formation fault recovery.

## 3. System Model

We divide UGV service deployment into initial static deployment and dynamic deployment. Static deployment aims to determine a service deployment strategy that, given the associations between UGVs’ processors and services, optimizes objectives such as minimizing typical service migration time and maintaining balanced processor loads within a single-vehicle environment, while also keeping the inter-vehicle migration ratio as small as possible. This paper performs dynamic service migration triggered by a processor failure in a multi-vehicle formation, based on an initial static deployment. Considering the impact of service image backups on the storage, memory, computing resources, and network bandwidth of UGVs, we adopt a certain proportion of backup images in our scheme.

UGV dynamic service migration includes hot migration and cold migration. In hot migration, the faulty vehicle performs state migration to a UGV processor that already hosts a backup image of the service; the service can resume operation by simply injecting the pending data from the faulty vehicle as the state. In cold migration, the service migrates to a processor without a corresponding service image backup, which requires both service image transfer [13] and state migration. For instance, over the inter-vehicle wireless link at the data rate of 200 kbps, cold-migrating a 10 MB service image requires 10 × 1024 × 8/200 ≈ 410 s, whereas hot-migrating a service by transferring only 8 KB of pending state data requires 8 × 8/ 200 ≈ 0.32 s.

**Figure 1:**
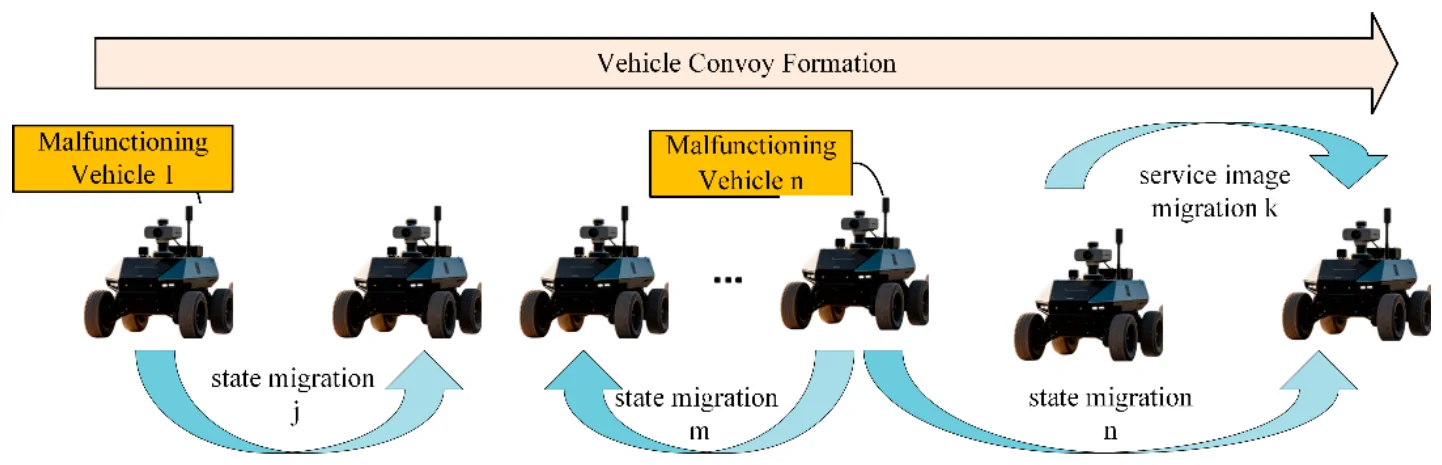
Physical communication links for UGV service migration.

The basic physical communication links for UGV dynamic service migration are as follows, as shown in Figure 1.

### 3.1. Migration Energy Cost Model

In the UGV formation communication environment, when a processor on a UGV fails, both the request for service migration and the transmission of original data required for post-migration service processing will affect the communication system power of the UGVs. We define the total transmission energy required to complete the migration of all failed services as the migration energy cost. Compared with the magnitude of wireless transmission energy consumption, the energy cost of service migration within a single vehicle is zero, as intra-vehicle communication runs on high-speed networks such as on-board Ethernet.

The wireless communication model in this paper is built on the following assumptions:

(i) Free-space path-loss propagation: We assume line-of-sight (LOS) links among UGVs and adopt the free-space path-loss model. Small-scale fading, shadowing, and multipath effects are neglected, which is acceptable because UGV formations operate over short-to-medium ranges with dominant LOS propagation.
(ii) Fixed modulation and coding scheme (MCS): The onboard wireless system adopts a fixed MCS and hence a constant data rate within the communication range. This rate is taken from the specifications of the wireless communication equipment actually mounted on the specific type of UGV considered in this study, and no rate adaptation to the instantaneous channel state is considered.
(iii) Constant received power: The onboard wireless system adjusts its transmit power to maintain a target received power, but the achievable transmit power is bounded by the strict constraints on the installation space and weight of the wireless system. Since the inter-vehicle distances of normal formation operations fall within the coverage range of this bounded transmit power, the target received power is always attainable and is therefore treated as a constant value.

We denote the service request and the amount of data to be transmitted from the faulty vehicle as *S* [13].

The total migration energy cost of the communication process is expressed as

*E_tx_* = *P_tx_* ⋅ *t* (1)

where *P_tx_* is the transmission power and *t* is the transmission time. Adopting the free-space path loss model and assuming a fixed data rate, the transmission power cost after service migration is related to the communication distance. This relationship is expressed as follows [22]:

*C_power_* = *γ* ⋅ *S* ⋅ *d*^2^ (2)

where *S* is the amount of transmitted data, *d* is the communication distance, and *γ* is a system constant.

#### 3.1.1. Derivation of the Power Cost Relationship for Wireless Communication

(a) Free-Space Path Loss Model

Under the free-space assumption, the received power is

(3) $$\begin{array}{c} P_{\mathrm{rx}}=P_{\mathrm{tx}}\cdot G_{t}G_{r}{\left(\frac{\lambda }{4\mathrm{\pi d}}\right)}^{2}\\ P_{\mathrm{rx}}=P_{\mathrm{tx}}\cdot \frac{K}{d^{2}} \end{array}$$

where *d* is the communication distance and *K* includes communication constants such as antenna gain and wavelength. To achieve the target received power $P_{\mathrm{rx}}^{\mathrm{target}}$, the required transmission power is

(4) $$P_{\mathrm{tx}}=P_{\mathrm{rx}}^{\mathrm{target}}\cdot \frac{d^{2}}{K}$$

(b) Transmission Energy

Based on a specific type of UGV wireless communication system, which adopts a fixed modulation and coding scheme with a data rate of *R*, the transmission time *t* is given by *t* = *S*/*R*.

Combining $P_{\mathrm{tx}}=P_{\mathrm{rx}}^{\mathrm{target}}\cdot \frac{d^{2}}{K}$ with *t* = *S*/*R*, we obtain

(5) $$E_{\mathrm{tx}}=P_{\mathrm{tx}}\cdot t=\left(\beta |d^{2}\right)\cdot \frac{S}{R}$$

where $\beta =P_{\mathrm{rx}}^{\mathrm{target}}/K$ absorbs all system constants.

Thus, we can derive

(6) $$E_{\mathrm{tx}}=\frac{\beta }{R}\cdot S\cdot d^{2}$$

From this, we conclude that the total energy for transmitting data after migration is proportional to the product of the data volume *S* and the square of the communication distance *d*^2^:

*C_power_* = *γ* ⋅ *S* ⋅ *d*^2^ (7)

where *S* is the total amount of data that needs to be transmitted after the service migration (in bits), and *d* is the distance between the vehicle executing the migrated service and the affected vehicle (in meters, computed via the Haversine formula), and γ is the energy coefficient (in J/(bit·m^2^)), calibrated to γ = 3.125 × 10^−9^ in our simulations. The calibrated value of γ is obtained from the system parameters of the specific UGV type.

The total service migration energy cost $C_{\mathrm{power}}^{\mathrm{total}}$ after a processor failure on a UGV is given by

(8) $$C_{\mathrm{power}}^{\mathrm{total}}=\sum_{m=1}^{M}\gamma \cdot S_{m}\cdot d_{m}^{2}$$

where *S_m_* is the amount of data that needs to be sent to the *m*-th vehicle receiving the migrated service, and *d_m_* is the distance between the faulty vehicle and the *m*-th target vehicle for service migration.

#### 3.1.2. Calculation of Onboard Communication Distance

UGVs are equipped with positioning devices. The distance calculation uses geographic coordinate information (longitude and latitude). We adopt the Haversine formula to calculate the spherical distance as the distance *d*(*CV_i_*, *CV_j_*) between UGVs [17]:

(9) $$\begin{array}{cc} & \phi_{1}=\mathrm{rad}(\mathrm{lat}({\mathrm{CV}}_{i})),\phi_{2}=\mathrm{rad}(\mathrm{lat}({\mathrm{CV}}_{j}))\\ & \eta_{1}=\mathrm{rad}(\mathrm{lon}({\mathrm{CV}}_{i})),\eta_{2}=\mathrm{rad}(\mathrm{lon}({\mathrm{CV}}_{j}))\\ & h=\sin^{2}\left(\frac{\phi_{2}-\phi_{1}}{2}\right)+\cos(\phi_{1})\cos(\phi_{2})\sin^{2}\left(\frac{\eta_{2}-\eta_{1}}{2}\right)\\ & c=2\cdot \arcsin(\sqrt{h})\\ & d\left({\mathrm{CV}}_{i},{\mathrm{CV}}_{j}\right)=R\cdot c\left(R\;=\;6371\;\mathrm{km}\right) \end{array}$$

### 3.2. Service Migration Time

When processors on a faulty UGV fail, the services running on it need to be migrated to other healthy processors. The migration process mainly consists of two steps: first, it ensures that the target processor has a backup of the corresponding service image and sends a service migration request; second, after the service request is acknowledged, the services continue executing the original tasks of the single vehicle to restore its normal functionality. The service migration time includes both the service image transfer time and the single-vehicle task execution time [23]:

*T* = *T_migr_* + *T_delay_* (10)

where the service image transfer time is denoted as *T_migr_*, and the single-vehicle task execution time as *T_delay_*.

The single-service migration delay *T_s_*_,*p*_ is given by

(11) $$T_{s,p}=\{\begin{array}{c} \frac{D_{\mathrm{image},s}}{B_{\mathrm{app}}},\;\;\;\mathrm{if}\;\mathrm{CV}\left(p\right)=\mathrm{CV}\left(s\right)\\ \frac{D_{\mathrm{image},s}}{R\left(d\left(p,\mathrm{fault}\right)\right)},\;\;\;\mathrm{if}\;\mathrm{CV}\left(p\right)\neq \mathrm{CV}\left(s\right)\; \end{array}$$

The single-service migration delay is determined by the link over which the service image is transferred: it uses the effective application-layer bandwidth of the intra-vehicle Gigabit Ethernet (*B_app_*) when the target processor is on the same vehicle, and the inter-vehicle wireless data rate (R(d)) when the target processor is on another vehicle, which reflects the orders-of-magnitude bandwidth difference between the two links.

Given that the wireless communication data rate between UGVs differs by orders of magnitude from the intra-vehicle Ethernet communication bandwidth, we simplify the *T_migr_* model in this paper. The calculation method is as follows:

(12) $$\begin{array}{c} T=\sum_{s\in S}\sum_{\mathrm{p\epsilon P}}x_{s,p}\cdot T_{s,p}\\ s.t.\;\sum_{p\in P_{\mathrm{local}}}x_{s,p}+\sum_{p\in P_{\mathrm{remote}}}x_{s,p}=1,\forall \;s\in S\\ x_{s,p}\in \{0,1\},\;\;\;\forall s\in S,\forall p\in P\\ \sum_{s\in S}x_{s,p}\leq C_{p},\forall p\in P \end{array}$$

*x_s_*_,*p*_ = 1 if *p* ∈ *S*_remote_; *x_s_*_,*p*_ = 0 if *p* ∈ *S*_local_. where *x_s_*_,*p*_ = 1 if service s is migrated to processor p, and *x_s_*_,*p*_ = 0 otherwise. The migration scheme must satisfy: (i) *x_s_*_,*p*_ is binary; (ii) the number of services assigned to processor p does not exceed its residual capacity *C_p_*, as shown in Table 1.

We define the task execution delay *T*_delay_ as the weighted cumulative sum of the edges along the execution path that the task flow traverses sequentially from the start node to the end node, passing through all service nodes. The calculation is given by

(13) $$T_{\mathrm{delay}}=\sum I_{s}^{T}\omega_{\mathrm{ij}}$$

where $I_{s}^{T}$ is the indicator vector between nodes, and *ω_ij_* is the weighted edge weight, which is related to the binary relations in the service graph and the volume of data interaction between services.

Considering the significant bandwidth difference between the intra-vehicle Gigabit Ethernet and the inter-vehicle wireless communication, and to accelerate the online computation of the algorithm, the execution delay between services deployed on the same vehicle is neglected. The mapping relationship between *ω_ij_* and the service deployment is given by formula (14):

(14) $$w_{\mathrm{ij}}=\{\begin{array}{c} 0,\;\;\exists m:v_{i}\in {\mathrm{CV}}_{m}\wedge v_{j}\in {\mathrm{CV}}_{m}\\ \frac{D_{\mathrm{ij}}}{R\left(d\left(\;\mathrm{CV}\left(p\right),\;\mathrm{CV}\left(s\right)\right)\right)},\;\;\;\;\mathrm{otherwise} \end{array}$$

where *v_i_* denotes the *i*-th node in the directed graph, and *D_ij_* is the volume of data interaction from node *v_i_* to *v_j_*.

The execution interaction between services typically carries a data volume on the order of a hundred bytes. Taking 100 bytes as an example, transmitting this data over the intra-vehicle Gigabit Ethernet (112.5 MB/s) takes only ≈ 0.9 µs, whereas over the inter-vehicle wireless link (200 kbps) it takes ≈ 4 ms, a difference of about 4500×. The execution delay between services deployed on the same vehicle is therefore negligible, and neglecting it does not affect the accuracy of the delay model.

A directed graph is constructed for all associated application services, and a DFS-based full-node traversal path search algorithm is adopted to determine the appropriate execution paths. The algorithm operates through a depth-first search with backtracking [24]. Starting from the source node, it recursively visits adjacent nodes along directed edges, thereby enumerating all loop-free paths. Whenever the target node is reached, the current path and its node count are recorded. After the traversal terminates, the algorithm selects the recorded path that contains the largest number of nodes, yielding the target full-node traversal path.

#### 3.2.1. Wireless Communication Rate Model

The calculation of migration energy cost in the system model involves the wireless communication rate *R*(*d*). Taking a specific type of UGV as an example, the wireless communication system adopts a fixed data rate. Given that the data rate *R*(*d*) is 200 kbps within this communication range.

#### 3.2.2. Ethernet Communication Rate Model

Ethernet communication is commonly adopted among onboard processors of UGVs. The actual available bandwidth, however, needs to account for the overhead introduced by protocol encapsulation at various layers.

Taking TCP and UDP protocols as benchmarks, the application-layer Ethernet communication rate is calculated as follows:

(15) $$R_{\mathrm{app}}=R_{\mathrm{line}}\times \frac{\mathrm{MTU}-H_{\mathrm{ip}}-H_{\mathrm{tcp}}}{\mathrm{MTU}+H_{\mathrm{mac}}+\mathrm{CRC}+P_{\mathrm{reamble}}+\mathrm{IFG}}$$

Considering the actual environmental losses, and assuming that the switch has non-blocking forwarding capability, the practical application-layer throughput accounts for approximately 90% of the total physical-layer transmission capacity. Consequently, the theoretical upper bound of the effective application-layer bandwidth is approximately 112.5 MB/s.

### 3.3. Load Balancing Rate

The load balancing rate is calculated using a weighted combination of CPU utilization, memory utilization, and network bandwidth utilization [14].

For a UGV processor *p*, the total resource demand required to host all services is given by

(16) $$\begin{array}{c} U_{p}^{\mathrm{cpu}}=\frac{\sum_{i=1}^{N_{\mathrm{FR}}}{1_{{(a}_{i}=p)}\cdot d}_{i}^{\mathrm{cpu}}}{C_{p}^{\mathrm{cpu}}}\\ U_{p}^{\mathrm{mem}}=\frac{\sum_{i=1}^{N_{\mathrm{FR}}}{1_{{(a}_{i}=p)}\cdot d}_{i}^{\mathrm{mem}}}{C_{p}^{\mathrm{mem}}}\\ U_{p}^{\mathrm{net}}=\frac{\sum_{i=1}^{N_{\mathrm{FR}}}{1_{{(a}_{i}=p)}\cdot d}_{i}^{\mathrm{net}}}{C_{p}^{\mathrm{net}}} \end{array}$$

where $U_{p}^{\mathrm{cpu}}$, $U_{p}^{\mathrm{mem}}$, and $U_{p}^{\mathrm{net}}$ denote the CPU, memory, and network resource utilization of processor *p*, respectively; $C_{p}^{\mathrm{cpu}}$, $C_{p}^{\mathrm{mem}}$, and $C_{p}^{\mathrm{net}}$ denote the total CPU, memory, and network resource capacities of processor *p*; $d_{i}^{\mathrm{cpu}}$, $d_{i}^{\mathrm{mem}}$, and $d_{i}^{\mathrm{net}}$ denote the CPU, memory, network resource demands of service *i*; ℱ*_R_* is the set of all services (including both normally running services and those to be migrated).

The load is computed by fusing the three types of resource utilization using a weighted average method, as follows:

(17) $$U_{p}^{\mathrm{comb}}=w_{\mathrm{cpu}}\cdot U_{p}^{\mathrm{cpu}}+w_{\mathrm{mem}}\cdot U_{p}^{\mathrm{mem}}+w_{\mathrm{net}}\cdot U_{p}^{\mathrm{net}}$$

where *w*_cpu_, *w*_mem_, and *w*_net_ denote the weights assigned to CPU, memory, and network resources, respectively, with *w*_cpu_ + *w*_mem_ + *w*_net_ = 1.

The load balancing rate *σ* is then expressed using the standard deviation:

(18) $$\sigma =\mathrm{std}\left(U_{p}^{\mathrm{comb}}\right)$$

### 3.4. Service Migration Coverage Rate

The service migration coverage rate *η* is defined as the ratio of the number of services that are successfully migrated to healthy processors to the total number of services that need to be migrated when a UGV processor fails:

(19) $$\eta =\frac{\sum_{\mathrm{s\epsilon S}}\mathrm{Success}\left(s\right)}{N_{\mathrm{total}}}$$

where *N*_success_ is the number of services successfully migrated, and *N*_total_ is the total number of services requiring migration.

The condition for a successful service migration is that there exists at least one processor *p* that satisfies the resource constraints.

(20) $$\mathrm{Success}(s)=\{\begin{array}{c} 1,\exists p\in P,\mathrm{Available}\left(p\right)\wedge x_{s,p}=1\\ 0,\mathrm{othersise} \end{array}$$

(21) $$\mathrm{Available}\left(p\right)=\{\begin{array}{c} 1,U_{p}^{\mathrm{cpu}}\leq \theta_{\mathrm{cpu}}\wedge U_{p}^{\mathrm{mem}}\leq \theta_{\mathrm{mem}}\wedge U_{p}^{\mathrm{net}}\leq \theta_{\mathrm{net}}\\ 0,\mathrm{othersise} \end{array}$$

where *θ*_cpu_, *θ*_mem_, and *θ*_net_ denote the CPU, memory, and network resource thresholds of processor *p*, respectively.

Each processor p has a normalized capacity of 100 on each resource dimension. The CPU, memory, and network demands of each service i are set with reference to the resource proportions occupied by the corresponding actual services of the specific UGV type. A migration scheme is feasible only if, after all services are placed, the utilization of each processor on each dimension does not exceed the corresponding threshold, i.e., *θ*_cpu_ = *θ_mem_* = *θ_net_* = 0.8.

## 4. Proposed Algorithm

Constrained by the complexity and uncertainty of operational scenarios, hardware faults in UGV formation scenarios exhibit stochastic characteristics. The dynamic service migration strategies for such scenarios are categorized into two types: intra-vehicle migration and inter-vehicle migration.

When a processor on a UGV fails, the migration mode—whether intra-vehicle migration or inter-vehicle migration—is guided by both resource availability and mission context. On one hand, the faulty vehicle’s healthy processors must have sufficient residual capacity (CPU, memory, and network) to cover all failed services. On the other hand, the formation’s current phase, e.g., engagement or assault windows, also plays a decisive role.

Let *S_f_* denote the set of failed services on the faulty vehicle, with resource demands $d_{s}=\;(d_{s}^{\mathrm{cpu}},d_{s}^{\mathrm{mem}}\;,\;d_{s}^{\mathrm{net}})$, and let *P_h_* denote its healthy processors with residual capacities $C_{p}\;=\;(C_{p}^{\mathrm{cpu}},\;C_{p}^{\mathrm{mem}},C_{p}^{\mathrm{net}})$. A fault is classified according to the following three criteria:

(a) Capacity criterion: The healthy processors of the faulty vehicle can collectively accommodate all failed services, i.e., $\sum_{s\in \;S_{f}}d_{s}^{r}\;\leq \;\sum_{p\in \;P_{h}}c_{p}^{r}\;$ for each resource *r* ∈ {*CPU*, *memory*, *network*}.
(b) Criticality criterion: The failed services are non-critical (i.e., outside the critical set that requires 100% backup).
(c) Mission-phase criterion: The current mission phase (e.g., non-engagement) does not require inter-vehicle load balancing.

A fault is classified as mild if all three criteria hold, in which case the intra-vehicle WOA strategy is invoked; otherwise, it is classified as severe, and the inter-vehicle SMAG-DQN strategy is invoked.

The decision rules of the mission-phase criterion are defined as follows:

(1) Transit/march phase—relaxed threshold, prefer intra-vehicle WOA migration. (a) Electromagnetic concealment: march usually requires low EM emission or silence; intra-vehicle migration is completed over onboard Ethernet without wireless leakage, whereas inter-vehicle migration may transmit megabyte-scale service images over wireless links and increase exposure risk. (b) Endurance: wireless transmission energy scales with the square of distance, so intra-vehicle migration consumes near-zero communication energy and preserves propulsion endurance. (c) Controlled failure risk: under the stable march load, the probability of cascading failure on the same vehicle is very low, and a local load imbalance only slightly slows non-critical services.

(2) Engagement/assault phase—tightened threshold, prefer inter-vehicle SMAG-DQN migration. (a) Vehicle survivability: concentrating all failed services on the remaining processors of the same vehicle risks total vehicle loss under a secondary fault, whereas inter-vehicle distribution realizes “one vehicle damaged, the formation takes over” redundancy. (b) Critical-service capacity: migrating non-critical services away reserves computing capacity for real-time critical services (navigation, situation awareness, motion control). (c) Cascading-failure hedging: shock, high temperature, and EMI in engagement significantly raise the probability of cascading processor failures, so tightening the load threshold disperses single-point risk across the formation. (d) Acceptable communication cost: engagement already involves high-frequency situation sharing and command interaction, so the added migration overhead is a small fraction of total traffic and is far outweighed by the cost of functional loss.

### 4.1. WOA-Based Intra-Vehicle Migration Strategy

For the intra-vehicle migration scenario, we employ the WOA. WOA is a swarm intelligence optimization algorithm that emulates the bubble-net hunting behavior of humpback whales to achieve global optimization [14]. The algorithm incorporates three position update mechanisms: encircling prey, bubble-net attacking, and random search. Among these, encircling prey and bubble-net attacking are responsible for local exploitation, while random search facilitates global exploration. The optimization objective is formulated as a weighted sum of normalized multiple performance metrics.

**Figure 2:**
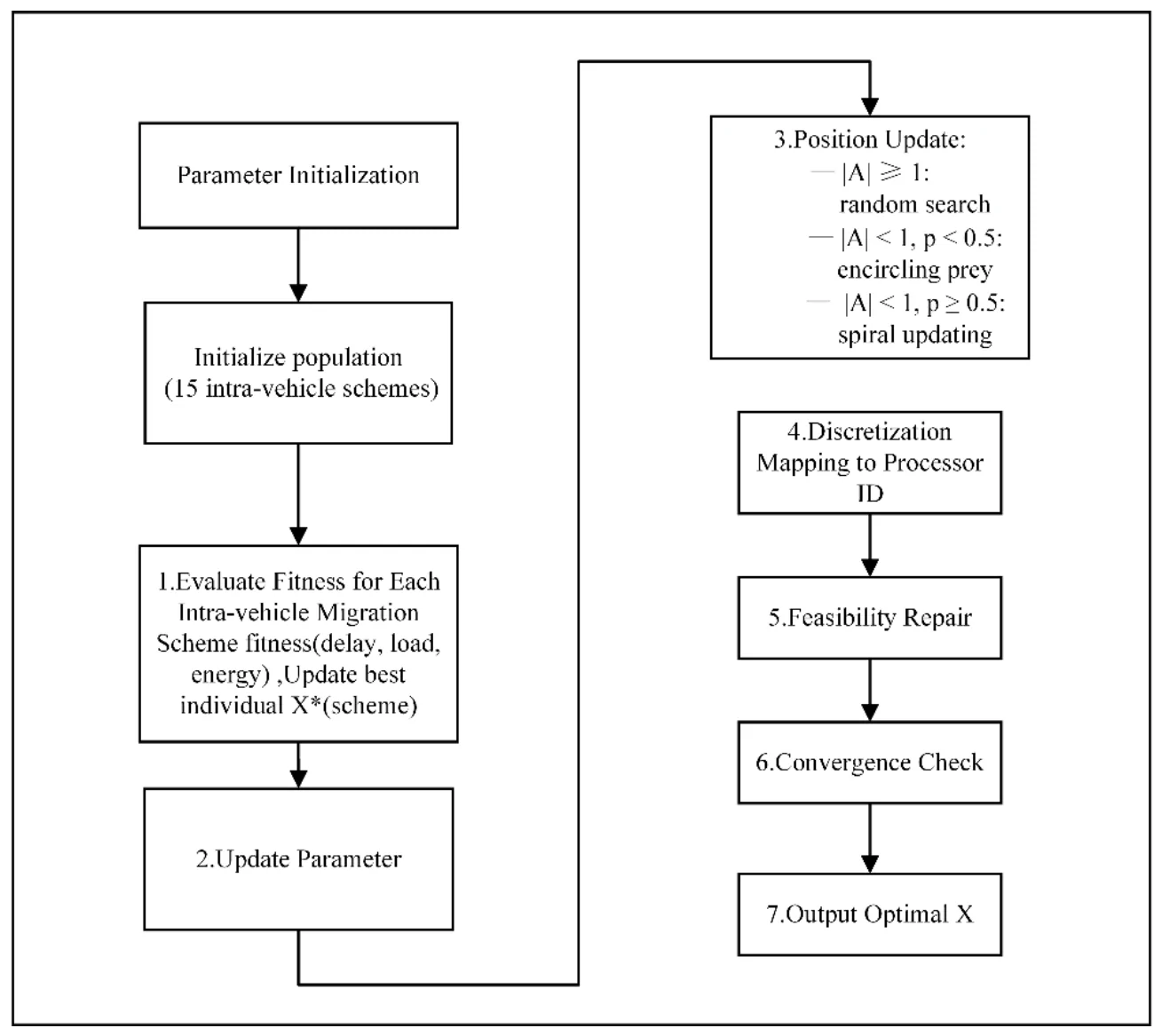
Block diagram of the WOA.

As shown in Figure 2, the WOA begins with parameter initialization and random generation of 15 candidate intra-vehicle migration schemes as the initial population. Each scheme is evaluated using a weighted fitness function that incorporates delay, load balancing, and energy consumption, and the best individual X* is identified. In each iteration, individuals update their positions via random search (|A| ≥ 1), encircling prey (|A| < 1, *p* < 0.5), or spiral updating (|A| < 1, *p* ≥ 0.5). The updated continuous positions are then discretized into processor IDs, followed by constraint repair for capacity and backup image feasibility to ensure the viability of service deployment. This iterative process continues until convergence, at which point the optimal migration scheme X and its corresponding delay and energy metrics are output.

### 4.2. SMAG-DQN-Based Inter-Vehicle Migration Strategy

For the inter-vehicle migration scenario, we propose a Spectral MAG-enhanced Adaptive DQN algorithm (SMAG-DQN) that synergistically integrates spectral clustering-based Migration Atomic Groups (MAGs) with the deep Q-network (DQN) framework. The underlying rationale is to retain DQN as the fundamental learning architecture while elevating the decision-making granularity to the MAG level. By exploiting the spectral structure embedded in the inter-service dependency matrix, we systematically uncover the underlying communication affinities among services, thereby biasing strongly coupled services toward co-migration to the same target processor. To further refine the migration policy, we augment the Q-network optimization with both cohesion and dispersion reward incentives, which jointly serve to reconcile the trade-off between preserving service association integrity and maintaining processor load balancing. Concretely, the spectral clustering-based MAG enhancement employs normalized spectral clustering to perform a topological decomposition of the service dependency graph [25], yielding a set of migration atomic groups (MAGs) that encapsulate services with pronounced topological interdependencies. Through the coordinated consideration of service instance aggregation patterns and resource capacity constraints, the proposed method facilitates service migration with high efficiency, low latency, and reduced energy expenditure.

#### 4.2.1. DQN Algorithm

**Figure 3:**
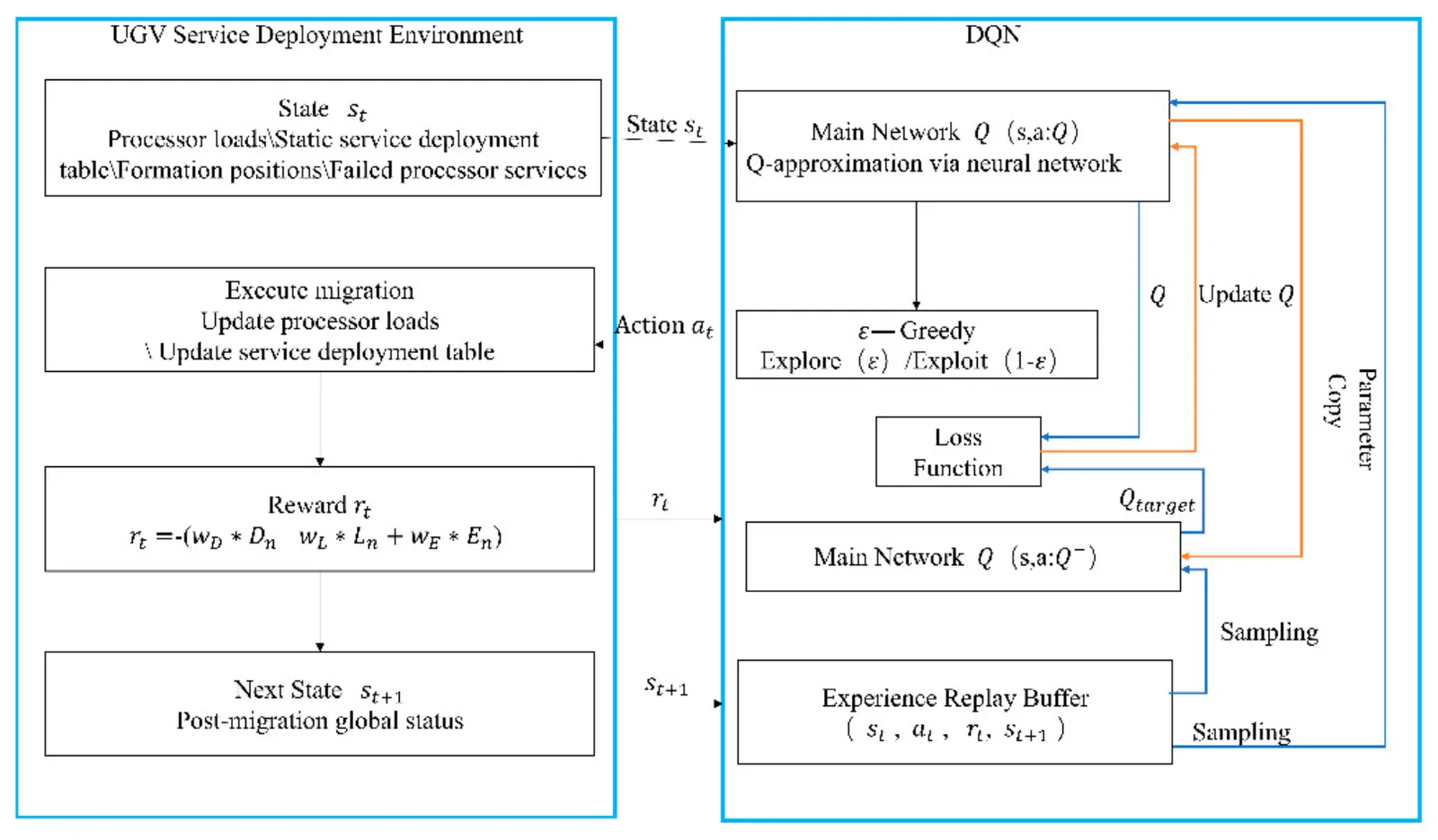
Framework of the DQN algorithm.

The DQN employs a deep neural network to approximate the action-value function *Q*(*s*, *a*; *θ*). The baseline DQN incorporates three key strategies—*ε*-greedy exploration, experience replay, and target network separation [26], which enable stable and efficient policy learning in complex environments. The framework of the DQN algorithm in the context of dynamic service migration for UGVs is illustrated in Figure 3. Our proposed SMAG-DQN algorithm builds upon this framework and introduces algorithmic enhancements tailored to the microservice migration scenario.

(a) Approximation of the Bellman Optimality Equation

The core of Q-learning is to learn an action-value function *Q*(*s*, *a*), which evaluates the expected cumulative discounted reward of taking action *a* in state *s*. This function is iteratively updated through the Bellman optimality equation:

(22) $$Q(s,a)\leftarrow Q(s,a)+\alpha \left[r+\gamma \cdot \underset{a^{\prime }}{\max}\;Q(s^{\prime },a^{\prime })-Q(s,a)\right]$$

where *α* is the learning rate that controls the step size of each update, and *γ* ∈ [0, 1] is the discount factor that balances the importance of immediate and future rewards.

Through the Temporal Difference (TD) method, the Q-value is incrementally corrected using the difference between the current estimate and the target value, namely, the TD error—and eventually converges to the optimal action-value function.

DQN employs a deep neural network *Q*(*s*, *a*; *θ*) as a function approximator, where *θ* denotes the network weights. The network takes the state feature vector as input and outputs the estimated Q-values for all feasible actions. The training objective is to minimize the mean squared error between the two sides of the Bellman optimality equation, i.e., to minimize the expected loss function of the TD error:

(23) $$L(\theta )=E\left[{\left(r+\gamma \cdot \underset{a^{\prime }}{\max}\;Q(s^{\prime },a^{\prime };\theta^{-})-Q(s,a;\theta )\right)}^{2}\right]$$

where *θ*^−^ denotes the parameters of the target network, which are employed to stabilize the generation of TD targets. The loss function *L*(*θ*) is optimized via gradient descent, enabling the network to gradually learn accurate Q-value estimates.

(b) ε-Greedy

Reinforcement learning involves both exploration and exploitation: exploration aims to discover unknown actions that may lead to superior policies, while exploitation leverages known high-value actions to accumulate rewards.

DQN adopts the ε-greedy policy as follows:

(24) $$a=\{\begin{array}{cc} \arg\max_{a}Q(s,a;\theta ), & 1-\varepsilon \\ \mathrm{random}\;\mathrm{action}, & \varepsilon \end{array}$$

In the early stages of training, the exploration rate is set to a relatively high value to thoroughly cover the state-action space. As training proceeds and the agent gradually learns reliable Q-value estimates, the exploration rate should be accordingly decayed to ensure policy convergence toward the deterministic optimal action. Specifically, we adopt an exponential decay schedule:

*ε* ← max(*ε*_end_,*ε* × *ε*_decay_) (25)

where the initial exploration rate is set to *ε*_start_ = 1.0 (i.e., complete random exploration), the decay factor is *ε*_decay_ = 0.99, and the lower bound is *ε*_end_ = 0.01 (i.e., retaining 1% random exploration to accommodate environmental variations). This decay mechanism ensures a dynamic balance between exploration and exploitation, accommodating both extensive exploration in the early training phase and policy convergence in the later phase.

(c) Experience Replay

The experience replay mechanism stores transition tuples (*s_t_*, *a_t_*, *r_t_*, *s_t_*_+1_) in a circular buffer of capacity *M* after each interaction with the environment. During training, a mini-batch of experiences is uniformly sampled from the buffer to compute gradients and update the network parameters.

The combination of experience replay and target network separation effectively mitigates the instability issues commonly encountered when training deep neural networks in reinforcement learning settings.

(d) Target Network Separation

In standard Q-learning, the TD target $y=r+\gamma \cdot \max_{a^{\prime }}Q(s^{\prime },a^{\prime };\theta )$ depends on the currently updated network parameters *θ*. This dependence causes the target value to change with each parameter update, resulting in a constantly shifting optimization objective that makes training highly prone to divergence.

DQN addresses this issue by maintaining two networks with identical structures but independently updated parameters: the main network *Q*_main_(*s*, *a*; *θ*) and the target network *Q*_target_(*s*, *a*; *θ*^−^). The main network is used for action selection and online parameter updates, while the target network is employed to compute the TD target $y=r+\gamma \cdot \max_{a^{\prime }}Q_{\mathrm{target}}(s^{\prime },a^{\prime };\theta^{-})$.

The parameters of the target network are periodically copied from the main network every *T*_update_ episodes (i.e., *θ*^−^←*θ*). Between two successive updates, the output of the target network remains fixed, thereby decoupling the computation of the TD target from the ongoing optimization of the current Q-values.

#### 4.2.2. Spectral Clustering-Based MAG Generation Algorithm

**Figure 4:**
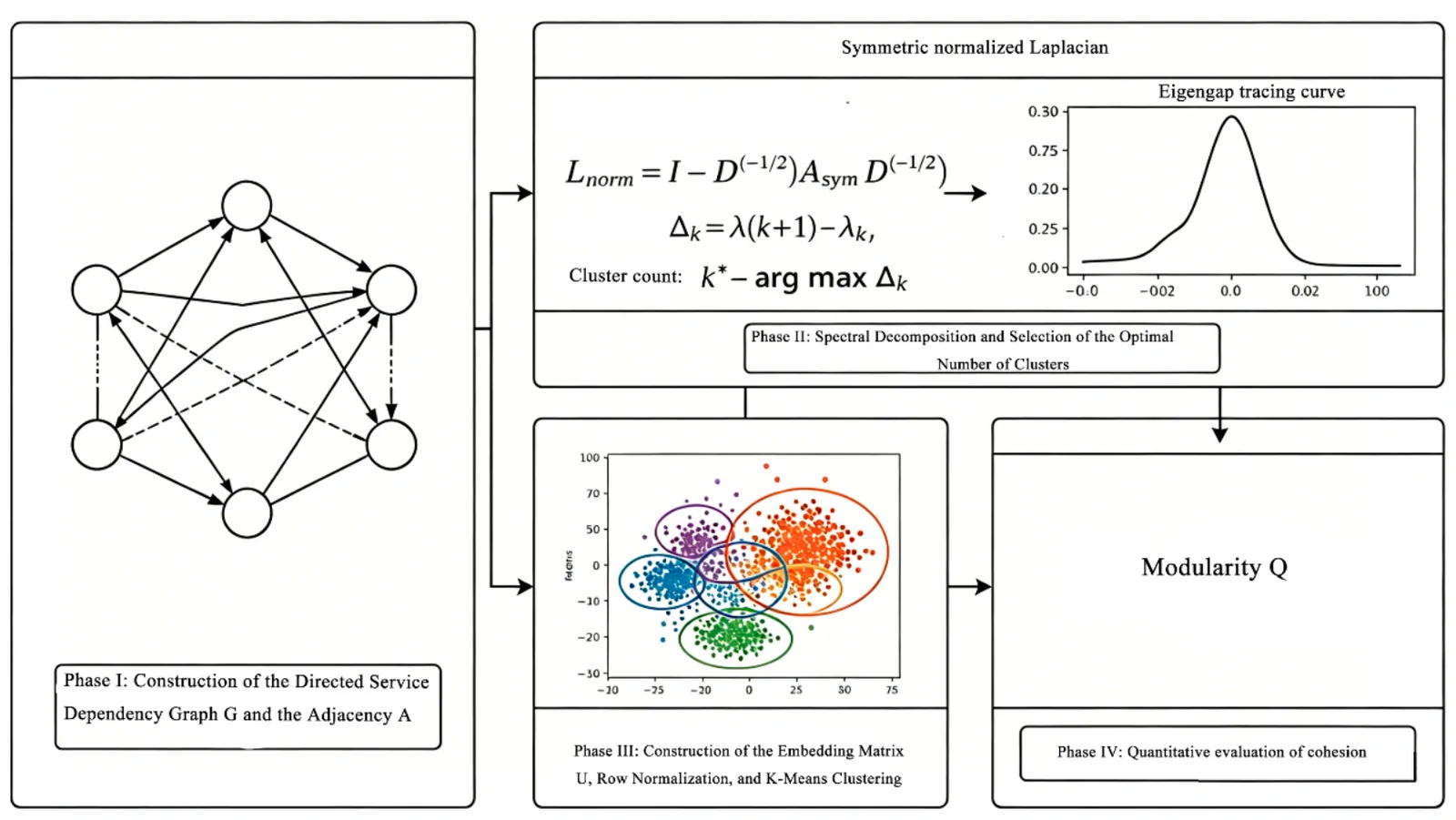
Spectral clustering-based MAG generation algorithm framework. In the scatter plot of Phase III, points with different colors represent different clusters obtained by spectral clustering.

Normalized spectral clustering [27] is applied to the grouping problem of the service dependency graph in UGV formations, which automatically aggregates microservices with communication dependencies into several migration atomic groups (MAGs) based on their affinity strengths. The process consists of four stages, and the detailed algorithmic flow is illustrated in Figure 4.

Phase I: We construct a directed service dependency graph *G* = (*V*, *E*) for the multiple microservices of UGVs, where *V* = {*s*_1_, *s*_2_, …, *s_m_*} corresponds to the individual microservices, and the edge set *E* denotes the communication data dependencies among services. By concatenating the service association matrices of the subsystems, we obtain an *m* × *m* adjacency matrix *A*.

Phase II: The symmetric normalized Laplacian matrix is constructed as follows:

*L*_norm_ = *I* − *D*^−1/2^*A*_sym_*D*^−1/2^ (26)

where *A*_sym_ is the symmetrized version of the adjacency matrix *A*, and *I* is the identity matrix. Eigenvalue decomposition is then performed on *L*_norm_, and the optimal number of clusters is determined by the eigengap heuristic:

(27) $$\Delta_{k}=\lambda_{k+1}-\lambda_{k},k^{*}=\arg\underset{k}{\max}\;\Delta_{k}$$

where *λ_k_* are the eigenvalues sorted in non-decreasing order. The optimal cluster number k^*^ is identified at the position where the eigengap Δ*_k_* attains its maximum.

Phase III: the first k^*^ non-trivial eigenvectors are extracted to construct the embedding matrix $U\in R^{m\times k^{*}}$, and each row of *U* is then normalized to have unit *l*_2_-norm. This operation projects the original service nodes onto a k^*^-dimensional unit hypersphere.

Phase IV: In the embedding space, K-means clustering with multiple random initializations is performed on all services. The partition that yields the maximum silhouette score is selected as the final clustering result, thereby obtaining k^*^ migration atomic groups (MAGs).

Within the same MAG, services exhibit tight dependencies, characterized by the parameter *ρ*_intra_; between different MAGs, dependencies remain sparse, characterized by the parameter *ρ*_inter_. The clustering quality is quantitatively evaluated by the modularity *Q* = *ρ*_intra_/*ρ*_inter_, which provides a structurally sound and cohesively coupled service aggregation metric for the subsequent SMAG-DQN migration decision algorithm.

#### 4.2.3. Algorithm Architecture

**Figure 5:**
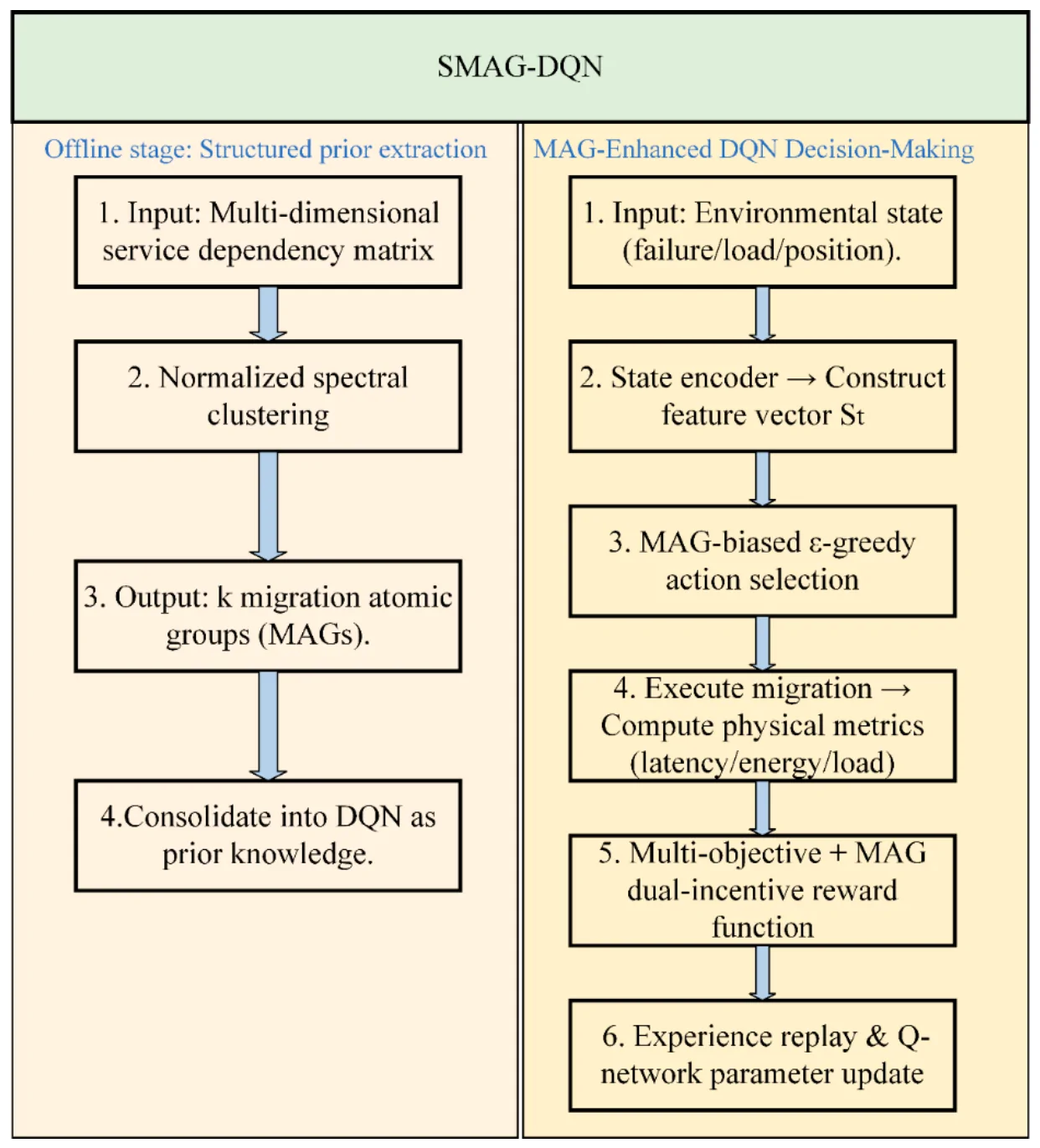
Framework of the SMAG-DQN algorithm.

The SMAG-DQN algorithm adopts a two-stage architecture consisting of “offline spectral clustering preprocessing + online MAG-biased enhanced DQN”, as illustrated in Figure 5.

In the offline stage, the algorithm performs normalized spectral clustering on the multi-dimensional service dependency matrix of UGVs to generate *k* migration atomic groups (MAGs), which serve as structured prior knowledge for the subsequent DQN-based reinforcement learning. In the online stage, both the MAG cohesion bias and the dispersion incentive are embedded into the *ε*-greedy action selection policy and the objective function, thereby guiding the algorithm to preferentially migrate services belonging to the same MAG to the same target processor.

#### 4.2.4. Offline Spectral Clustering Preprocessing Stage

In the offline spectral clustering preprocessing stage, we adopt an offline MAG generation strategy based on normalized spectral clustering. This process is performed once offline prior to the start of training. First, the services on the faulty processors of UGVs are identified, and the faulty services with dependencies among them are partitioned into *k* migration atomic groups (MAGs), maximizing the intra-group dependency strength among services. The specific implementation is as follows:

Step 1: Symmetrize the adjacency matrix of the directed service dependency graph and construct the Laplacian matrix.

Given the adjacency matrix *A* ∈ R*^N^*^×*N*^ of the directed service dependency graph, we first symmetrize the asymmetric directed adjacency matrix as

(28) $$A_{\mathrm{sym}}=\frac{1}{2}(A+A^{T})$$

We then construct the diagonal degree matrix *D* = diag(*d*_1_, *d*_2_, …, *d_N_*), where the degree of node *i* is defined as

(29) $$d_{i}=\sum_{j=1}^{N}A_{\mathrm{sym}}(i,j)$$

the symmetric normalized Laplacian matrix is generated as

(30) $$L_{\mathrm{norm}}=I-D^{-\frac{1}{2}}{*A}_{\mathrm{sym}}*D^{-1/2}$$

Step 2: Eigenvalue decomposition is performed on the normalized Laplacian matrix *L*_norm_, and the first *k*_max_ smallest eigenvalues are extracted. The eigengap sequence is then constructed as

∆*_k_* = *λ_k_*_+1_ − *λ_k_*, *k* = *k_min_*,…, *k_max_* (31)

According to matrix perturbation theory, the value corresponding to the maximum eigengap yields the optimal number of clusters k^*^ for the service graph:

(32) $$k^{*}=\arg_{k\in [k_{\min},\;k_{\max}]}\max{∆}_{k}$$

Step 3: The eigenvectors corresponding to the first *k*∗ smallest non-zero eigenvalues of *L*_norm_ are extracted to form the embedding matrix $U\in R^{N\times k^{*}}$ (discarding the trivial eigenvector associated with *λ*_1_ = 0). The rows of *U* are then 𝓁_2_-normalized as

(33) $$U_{i}^{\mathrm{norm}}={\frac{U_{i}}{\;||U_{i}||}}_{2}\;i=1,2,\ldots ,N$$

In the normalized embedding space, K-means clustering is performed on the *N* services, with the process repeated 15 times and the silhouette coefficient adopted as the selection criterion to identify the optimal partition. The MAG assignment vector, which records the cluster label of each service, is produced as the output.

Step 4: To quantitatively evaluate the quality of the generated MAG partitions, two metrics are introduced: the intra-MAG dependency density *ρ*_intra_ and the inter-MAG dependency density *ρ*_inter_. These metrics are defined as

(34) $$\begin{array}{c} \rho_{\mathrm{intra}}=\frac{\sum_{g=1}^{k^{*}}\sum_{\begin{array}{c} j\in G_{g}\\ i\neq j \end{array}}A_{\mathrm{sym}}(i,j)}{\sum_{g=1}^{k^{*}}n_{g}(n_{g}-1)}\\ \rho_{\mathrm{inter}}=\frac{\sum_{g\neq h}\sum_{\begin{array}{c} i\in G_{g}\\ j\in G_{h} \end{array}}A_{\mathrm{sym}}(i,j)}{\sum_{g\neq h}n_{g}n_{h}} \end{array}$$

where *ρ*_intra_ is the intra-MAG dependency density and *ρ*_inter_ is the inter-MAG dependency density.

#### 4.2.5. Online Training Stage

During the online training stage, the MAG structural information generated in the offline stage is integrated into the action selection mechanism of DQN. The Q-value estimation is calibrated by introducing both a MAG cohesion bias and a MAG dispersion incentive, which collectively guide the agent to preserve MAG cohesion during both exploration and exploitation phases.

The online training stage of the SMAG-DQN algorithm incorporates the following four enhancements to the classical DQN framework: adaptive boundary rescaling, MAG-guided action space compression, hybrid TD learning with MAG-structure rewards, and structured bias injection into Q-value ranking. The training parameters, including exponential decay of *ε*, hard update period of the target network, and stepwise decay of the learning rate, follow the standard configurations of classical DQN.

(a) Multi-Objective Optimization Reward

The total reward for service migration consists of four components: the objective optimization reward *R*_opt_, the constraint penalty *R*_pen_, the MAG cohesion reward *R*_co_, and the MAG dispersion incentive reward *R*_di_. The computation of *R* is as follows:

*R* = *R*_opt_ − *R_pen_* + *R_co_* + *R_di_* (35)

At the beginning of each training episode, the extreme value ranges of the three optimization objectives are computed through global simulation, and a dynamic normalization reference baseline is established. Specifically, for migration delay *T*_delay_, migration energy cost *E*, and load balancing rate *σ*, a greedy algorithm is employed to traverse all full combinations of the services to be migrated across all non-faulty processors, thereby obtaining the global minimum and maximum values for each objective. Based on these adaptive extrema, each objective is normalized to the interval [0, 1]. The objective optimization reward *R*_opt_ is then computed as

*R*_opt_ = −*ω_D_D_norm_* − *ω_N_N_norm_* − *ω_E_E_norm_* (36)

(b) MAG Cohesion Bias

Our method introduces a MAG cohesion bias to guide the agent toward prioritizing the migration of services to nodes that already host members of the same MAG, thereby ensuring that tightly coupled services are deployed in proximity.

For a service *s_i_* to be migrated, which belongs to $G_{g_{i}}$, if the candidate target processor *p_v_*_,*j*_ (the *j*-th processor on UGV *v*) already hosts one or more members of the same MAG, a positive bias is added to the Q-value:

∆*Q_coh_*(*s_i_*, *p_v_*_,*j*_) = *ω_c_*|*G_gi_* ∩ *D*(*v*, *j*)| (37)

where 𝒟(*v*, *j*) denotes the set of services currently deployed on processor *p_v_*_,*j*_, and *ω_c_* is the cohesion weight coefficient.

(c) MAG Dispersion Incentive Bias Configuration

When an inter-vehicle migration is performed, if the source vehicle still hosts unmigrated services belonging to the same MAG, a small positive incentive is granted to the inter-vehicle action. Conversely, if intra-vehicle migration is selected, a penalty is imposed based on the load difference. The MAG dispersion incentive bias is computed as follows:

(38) $$∆Q_{\mathrm{disp}}\left(s_{i},\;p_{v,j}\right)=\left\{\begin{array}{c} \omega_{d}\left|G_{\mathrm{gi}}\cap \;D\left(v_{\mathrm{src}},\;p\right)\right|,v\neq v_{\mathrm{src}}\\ -\mu {*\omega }_{d}*\max\left(0,L_{v,j}^{,}-{\bar{L}}_{v,-j}\right),v=v_{\mathrm{src}} \end{array}\right\}$$

where *v*_src_ is the faulty source vehicle; $L_{v,j}^{,}$ is the predicted load of the target processor after migration completion; ${\bar{L}}_{v,j}$ is the average load of the other healthy processors on the same vehicle; *ω_d_* is the dispersion weight coefficient; and *μ* is the load penalty factor.

(d) MAG-Enhanced ε-Greedy Strategy

The MAG cohesion bias and MAG dispersion incentive bias are incorporated into the Q-value to construct the MAG-enhanced Q-value:

(39) $$\tilde{Q}(s_{t},\;a)\;=\;Q(s_{t},\;a;\;\theta )\;+\;{∆Q}_{\mathrm{coh}}(s_{i},\;a)\;+{∆Q}_{\mathrm{disp}}(s_{i},\;a)$$

Furthermore, the ε-greedy action selection strategy is modified as follows: with probability ε, an action is uniformly and randomly sampled from the set of valid target processors; with probability 1 − *ε*, the action corresponding to the maximum MAG-enhanced Q-value is selected:

(40) at={Random(Avalid),ξ<ε arg maxQ~(st, a) ,ξ≥ε}

where *A*_valid_ denotes the set of valid target processors that satisfy the capacity constraints, and *ξ*∼*U*(0, 1).

## 5. System Simulation

### 5.1. Simulation Setup

We take a formation composed of a specific type of UGV as the research object. The overall vehicle service relationship is used as the database, which is stored in the form of a multi-dimensional Boolean matrix. Services constitute the element set of the matrix, and if an association exists between two elements, the corresponding entry is assigned a value of 1; otherwise, it is 0. This approach quantifies the interaction relationships among services.

**Figure 6:**
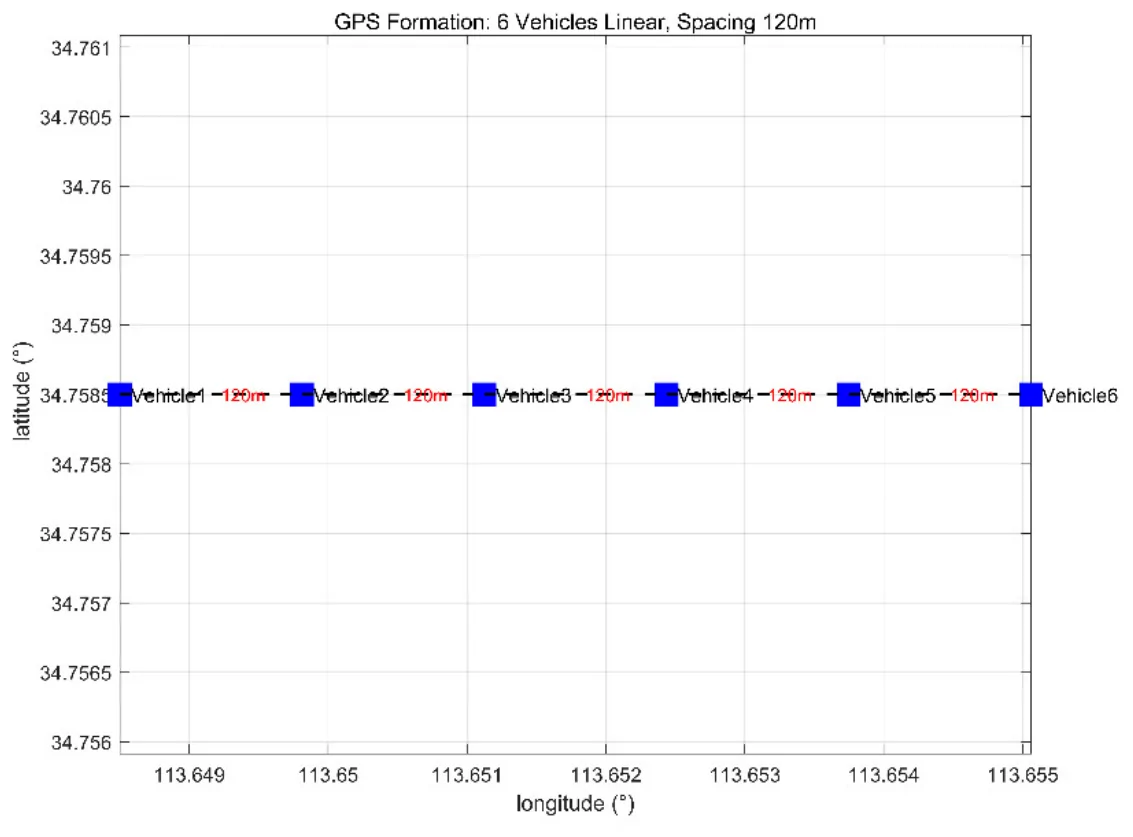
Geographic positions of UGVs in one-line formation.

**Figure 7:**
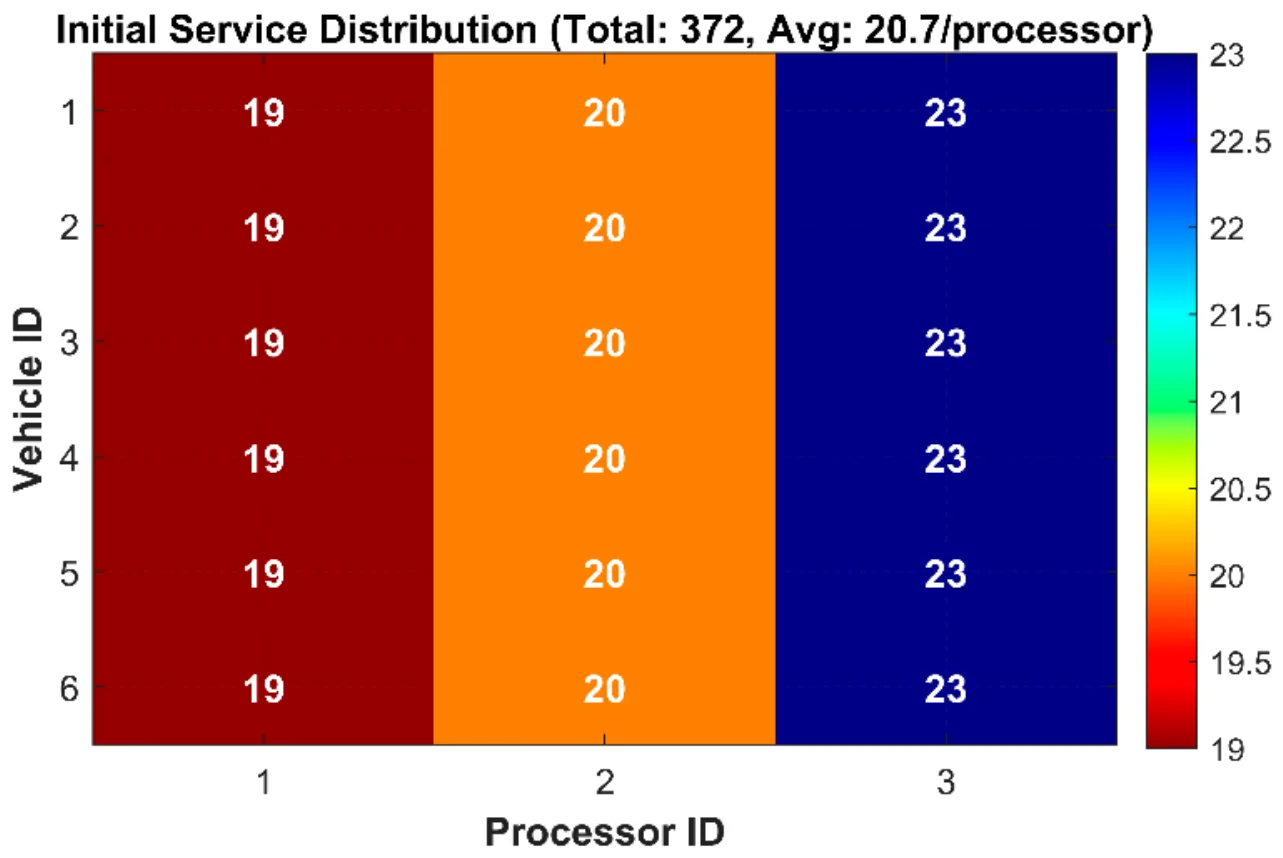
Static service deployment of UGV formation.

**Figure 8:**
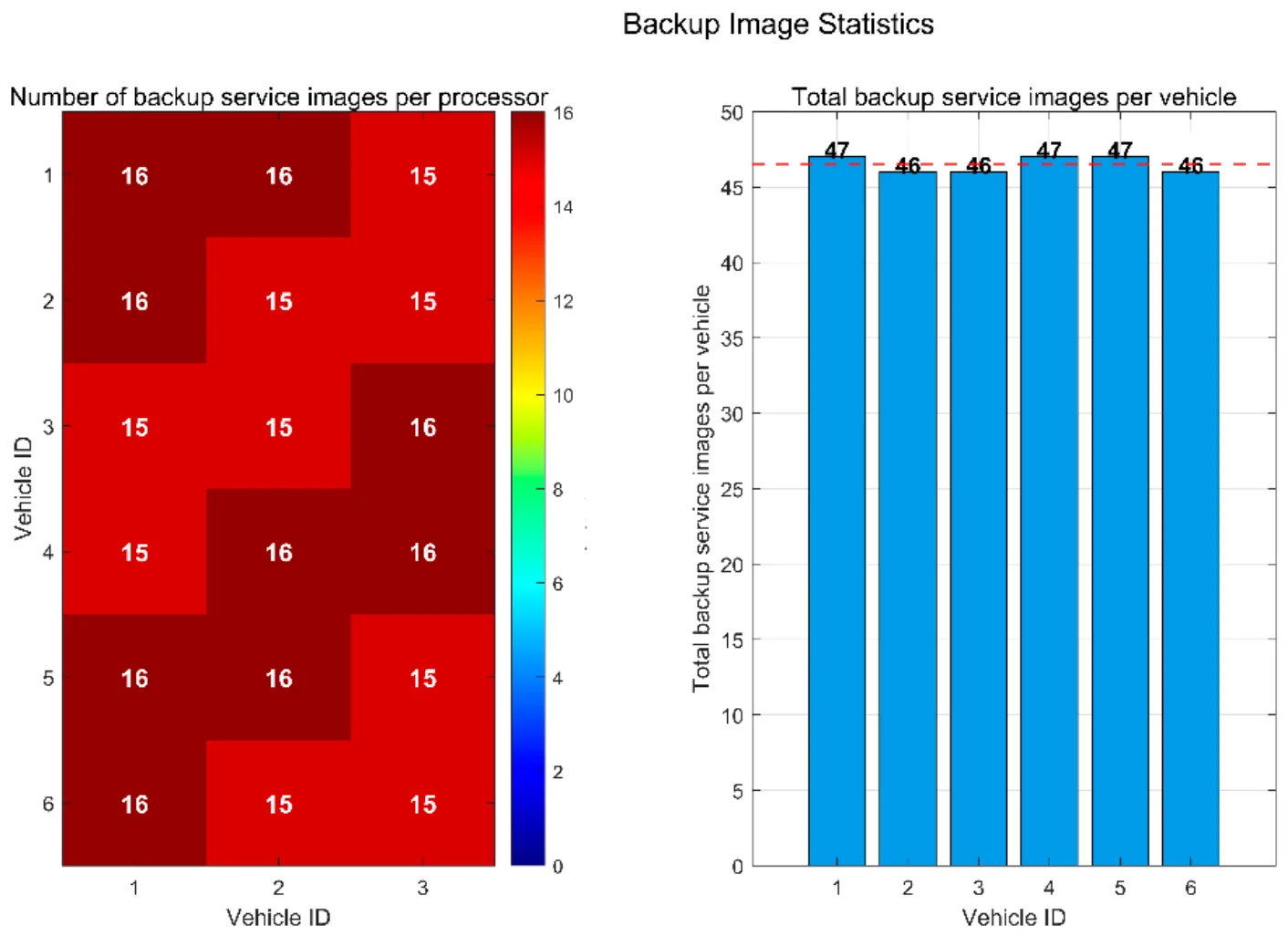
Image backup distribution on each vehicle in the formation.

According to the formation pattern during UGV movement and engineering practice requirements, a typical one-line formation is adopted. Figure 6 shows the geographic positions of the one-line formation, and Figure 7 illustrates the static deployment of processors on each vehicle in the formation. The onboard autonomous driving system employs 100% backup for critical services, while the remaining services are backed up at a ratio of 50% of the total number of vehicles in the formation. The backup status of processors on each vehicle is shown in Figure 8.

We explicitly configure the fault scenario by specifying the set of failed processors as (vehicle, processor) pairs. In the reported simulations, processors 1–2 of vehicle 1, processor 1 of vehicle 2, and processor 2 of vehicle 3 fail simultaneously, which covers both the case of multiple processors failing concurrently on a single vehicle and the case of processors failing on multiple vehicles. The failed processors remain unavailable throughout the migration episode.

### 5.2. Simulation Results and Analysis

#### 5.2.1. Simulation Analysis of WOA-Based Intra-Vehicle Migration

**Figure 9:**
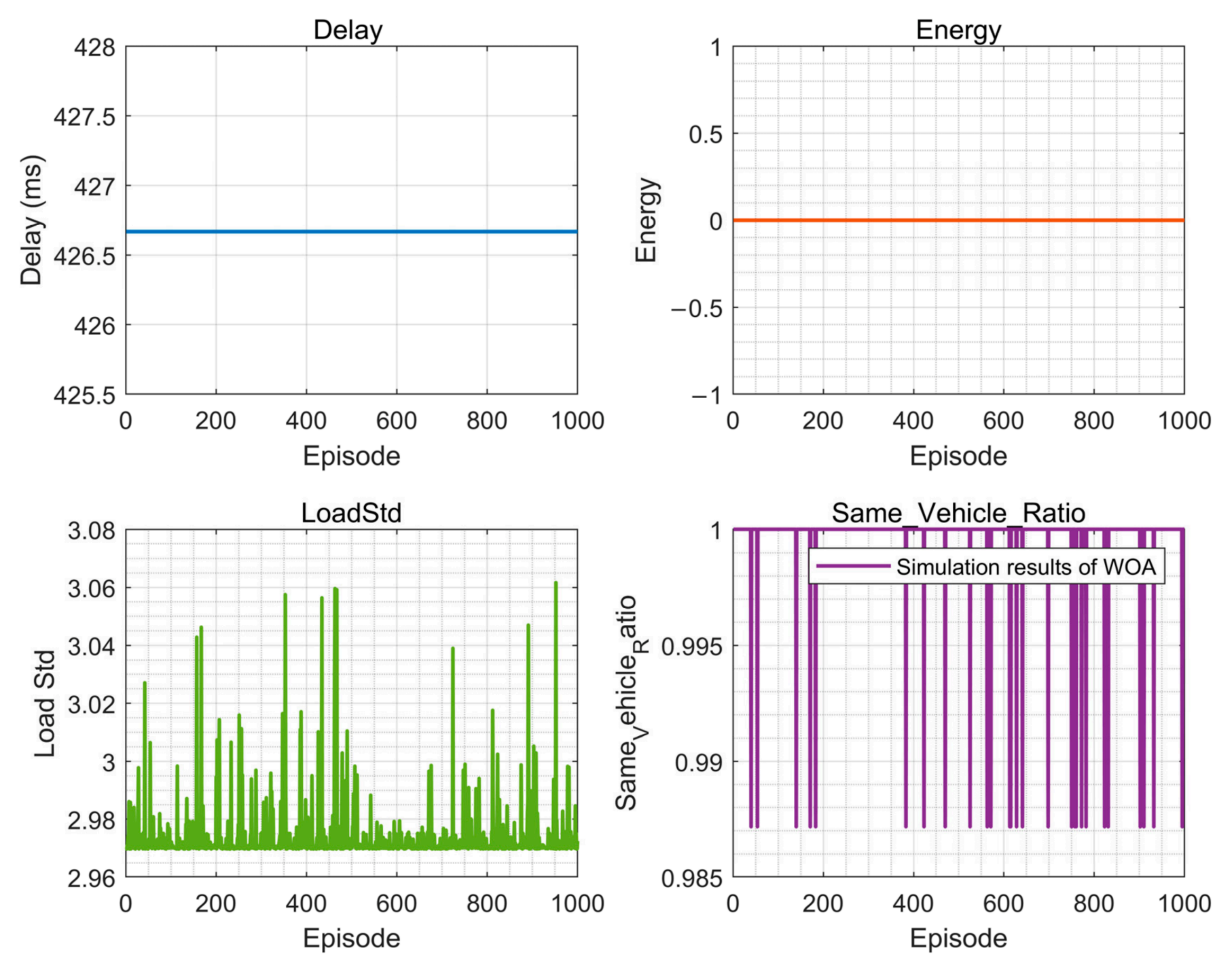
Performance of WOA-based intra-vehicle migration.

**Figure 10:**
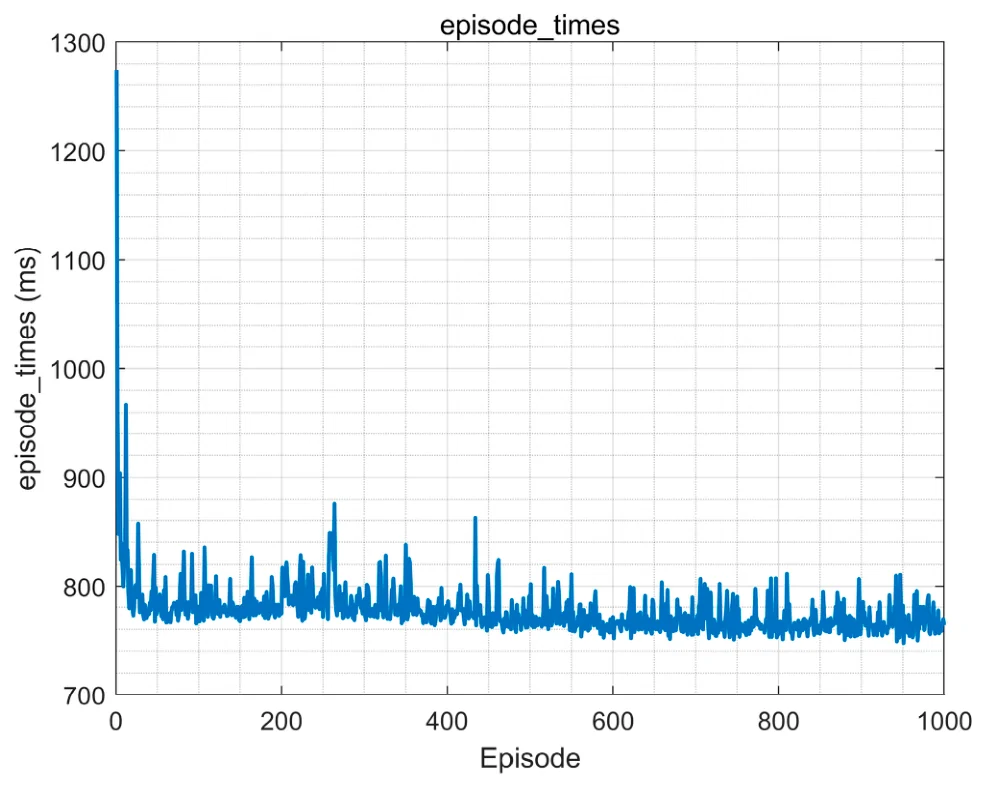
Per-episode computation time of the WOA-based intra-vehicle migration.

For the WOA-based intra-vehicle migration strategy, the population size is set to 15 and the number of iterations to 20, with a total of 1000 repeated training episodes. As shown in Figure 9 and Figure 10, all services requiring migration are successfully transferred within the same vehicle, with no inter-vehicle task migration occurring. Consequently, the migration energy cost remains approximately 0 J. The migration success coverage rate attains 100%, and the average computation time per episode is only 0.78 s. The load balancing rate, measured by the standard deviation of processor loads, remains approximately 2.97, indicating that the entire workload from the failed processors is intensively offloaded onto the limited number of remaining healthy processors within the same vehicle.

#### 5.2.2. Simulation Analysis of SMAG-DQN-Based Inter-Vehicle Load Balancing Migration

The SMAG-DQN-based inter-vehicle load balancing migration algorithm is trained for 1000 episodes and subsequently evaluated through 10 independent runs. We have consolidated the key algorithm parameters into Table 2.

**Figure 11:**
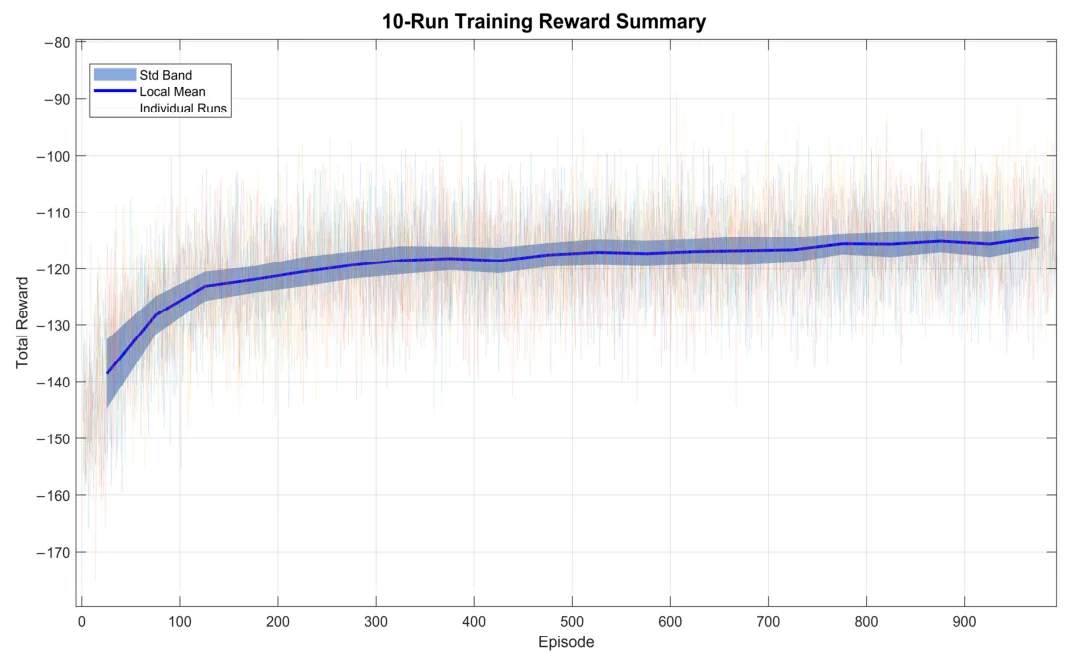
Training reward curves.

Reward curves averaged over the 10 independent runs are shown in Figure 11, where a sliding window is applied for local mean smoothing. As observed from Figure 11, the algorithm exhibits rapid exploration and learning in the early stage, fine-grained optimization in the middle stage, and a stable convergence plateau in the later stage, which is consistent with the convergence characteristics of reinforcement learning algorithms.

Based on simulation experiments with 10 independent runs for both SMAG-DQN and baseline DQN, a systematic comparative analysis is conducted from the aspects of policy quality, mechanism ablation, convergence and robustness, and computational efficiency, to evaluate the impact of introducing clustering information.

(a) Effect Analysis of Introducing the Clustering Mechanism

**Figure 12:**
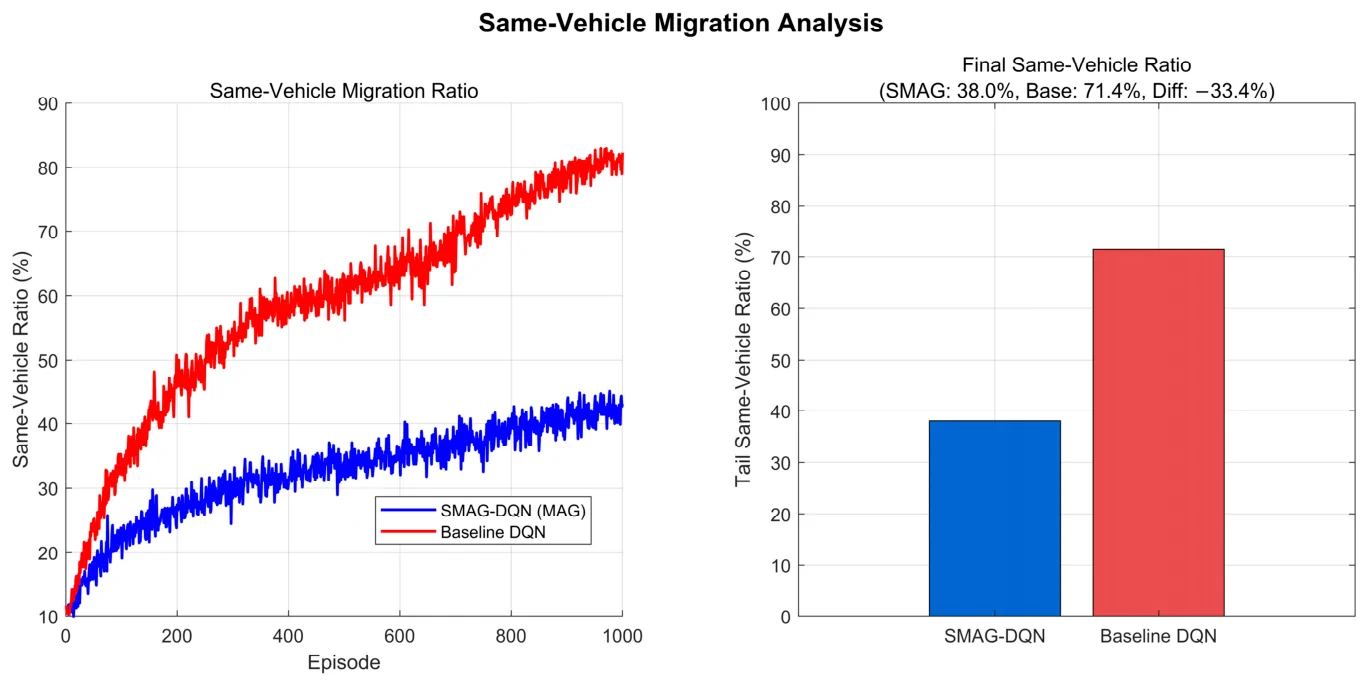
Same-vehicle migration rate.

As shown in Figure 12, the intra-vehicle migration rates of SMAG-DQN and baseline DQN are 38.0% and 71.4%, respectively. Baseline DQN tends to migrate services to other processors on the same vehicle, whereas SMAG-DQN converts 33.4% of migrations from intra-vehicle to inter-vehicle to distribute the load across the entire formation. In each migration, if the target vehicle ID is the same as the source vehicle ID, it is counted as an intra-vehicle migration. The intra-vehicle migration rate is defined as the ratio of intra-vehicle migrations to the total number of migrations.

**Figure 13:**
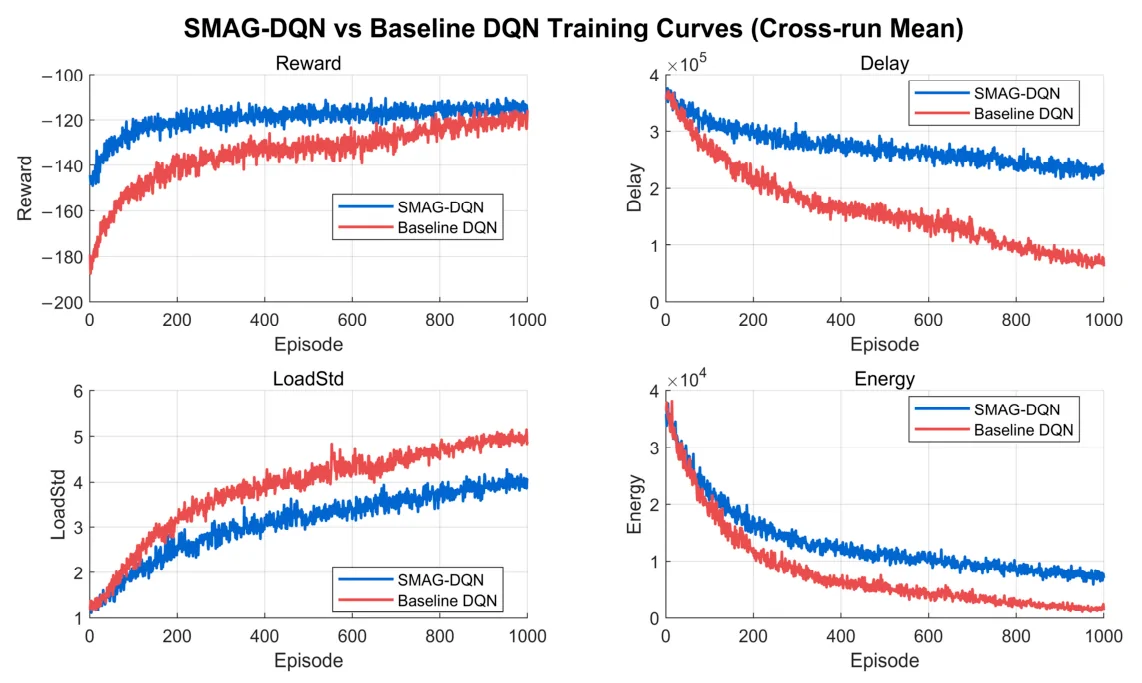
Objective function simulation results of SMAG-DQN and DQN.

Figure 13 presents the simulation results for load balancing rate, migration delay, and migration energy cost. The averaged values of these metrics are taken after the total reward stabilizes, and the results are summarized in Table 3. SMAG-DQN improves the total reward from −125.84 to −116.11, achieving a 7.7% enhancement, with a standard deviation of ±2.33 across the 10 runs, which is smaller than that of baseline DQN (±5.04). This demonstrates the effectiveness of incorporating the clustering prior knowledge of microservice application groups (MAGs) into the reward function.

(b) Convergence and Robustness Analysis

The coefficient of variation (CV) is adopted as the evaluation metric. The data are taken from 500 episodes after the reward stabilizes.

(41) $$\mathrm{CV}=\frac{\beta }{\alpha }$$

where *α* denotes the mean values of the load balancing rate, migration delay, and migration energy cost, and *β* denotes their corresponding standard deviations.

As shown in Table 4, the CV of reward and latency for SMAG-DQN is 5.7% (compared with 25.7% for baseline DQN), and the CV of migration energy cost is also lower than that of baseline DQN. Compared with the fluctuation data in Table 4, SMAG-DQN reduces the performance fluctuation across multiple runs by more than 50%. The latency CV of baseline DQN reaches 25.7%, indicating that the cumulative error of per-service decision-making makes the algorithm highly sensitive to random seeds. By contrast, SMAG-DQN compresses the decision chain from 78 steps to approximately 15–20 steps through MAG-level batch decision-making, effectively suppressing error propagation.

The analysis shows that the MAG clustering mechanism compresses the number of decision steps, significantly reducing the accumulation path of Q-value errors, thereby mitigating the influence of random seeds on the final policy and enhancing the determinacy of the algorithm. Under the same random seed perturbations, the policy output of SMAG-DQN remains more stable.

(c) Computational Efficiency Analysis

**Figure 14:**
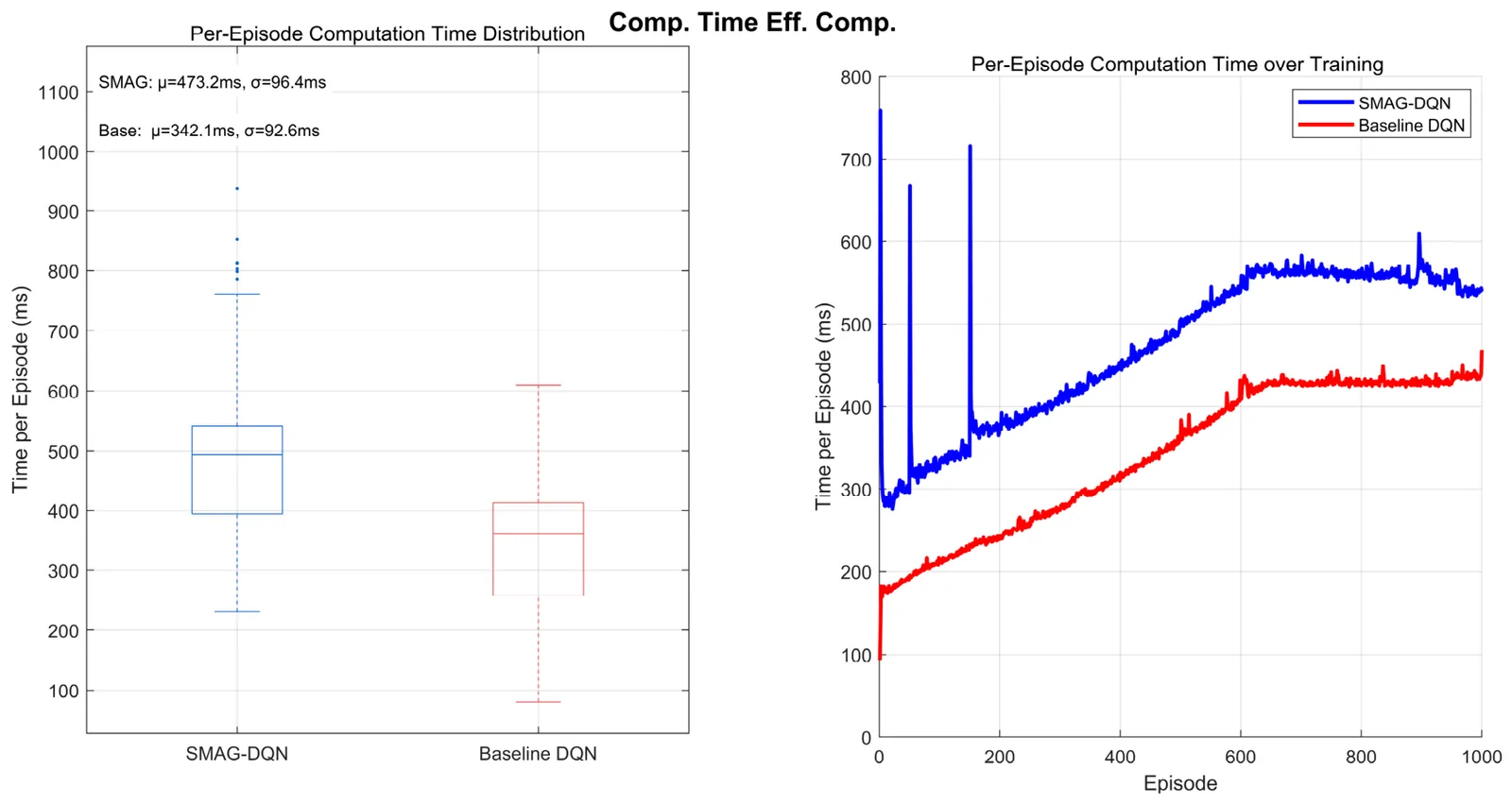
Average computation time per-episode (10-run average).

As shown in Figure 14, the computation time of SMAG-DQN remains consistently higher than that of baseline DQN throughout the entire process, with noticeable fluctuations in the initial initialization phase. The reason is that although the number of decision steps is compressed from 78 steps to approximately 15–20 steps, the preprocessing overhead per step increases significantly. The whole-group capacity pre-check requires traversing all candidate target nodes and simulating the feasibility of simultaneous migration of all services within a MAG. Moreover, the computation of cohesion and dispersion biases requires repeated statistics on the distribution of the same MAG across different nodes, which increases the per-step computational complexity and thus offsets the benefits gained from the reduction in the number of steps.

### 5.3. Algorithm Comparison and Validation

To explore the applicability of different algorithms for dynamic migration of faulty services in cooperative UGV formations, we select two algorithms as benchmarks: NSGA-II algorithm, and a group migration algorithm based on Gibbs sampling [5] and Lyapunov optimization. These algorithms serve as typical representatives in the fields of heuristic strategies and mathematical optimization, respectively.

Comparison of NSGA-II

The genetic algorithm is a global optimization method that mimics the process of natural evolution, iteratively searching for the optimal solution in the solution space through three basic operations: selection, crossover, and mutation [7]. The encoding scheme adopts an integer representation where each gene corresponds to the target processor ID assigned to a service instance to be migrated, with the chromosome length equal to the total number of instances requiring migration under the current fault scenario. The fitness function is consistent with that of DQN, defined as the normalized weighted sum of migration delay, load balancing rate, and migration energy cost, with hard constraint penalties included.

Comparison of Group Migration Algorithm Based on Gibbs sampling and Lyapunov Optimization

Lyapunov optimization originates from stochastic network optimization theory and was initially developed to address the trade-off between stability and performance in wireless communication and queue scheduling problems. Its core idea is to construct a Lyapunov function (typically the sum of squares of queue lengths) and steer the system state toward a stable region by minimizing the conditional expectation of the drift of this function. In the service migration scenario, migration delay, load balancing rate, and migration energy cost are defined as three system state variables. A penalty factor is introduced to quantify the deviation of each metric from its target value, and the Lyapunov drift serves as the core term of the energy function, guiding decisions toward simultaneously satisfying multiple constraints. Gibbs sampling is employed for target selection, with a simulated annealing mechanism used to dynamically adjust the temperature parameter, balancing exploration and exploitation.

The above algorithms are all evaluated through simulation experiments over 1000 episodes, among which NSGA-II is additionally conducted with 10 independent runs. All simulations are performed on the MATLAB R2022b platform. The algorithm performance is compared and analyzed from the following four aspects: load balancing performance, task migration characteristics, computational rapid performance, and migration delay and migration energy cost.

#### 5.3.1. Multi-Dimensional Performance Comparison

**Figure 15:**
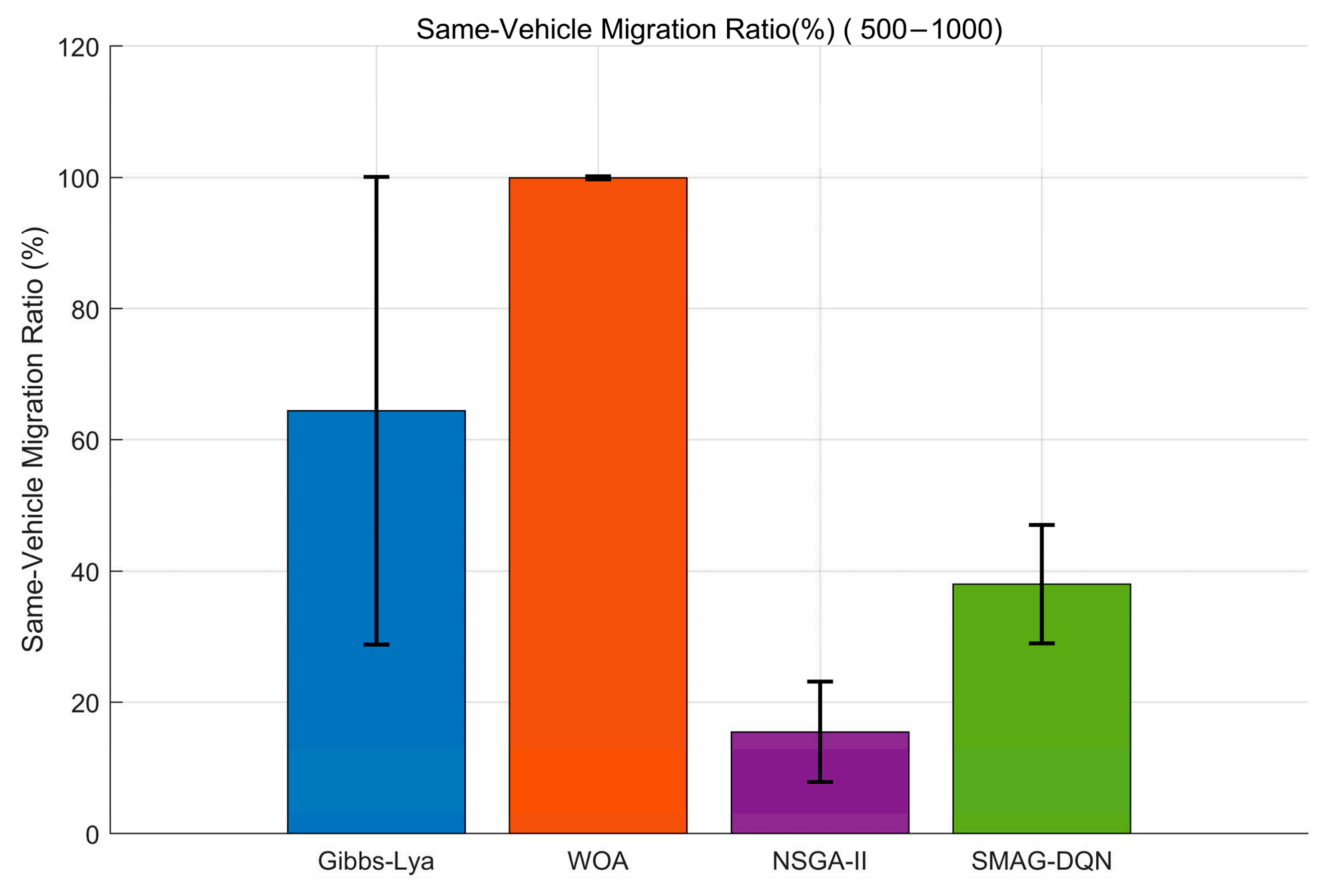
Comparison of Same-vehicle Migration Ratio.

**Figure 16:**
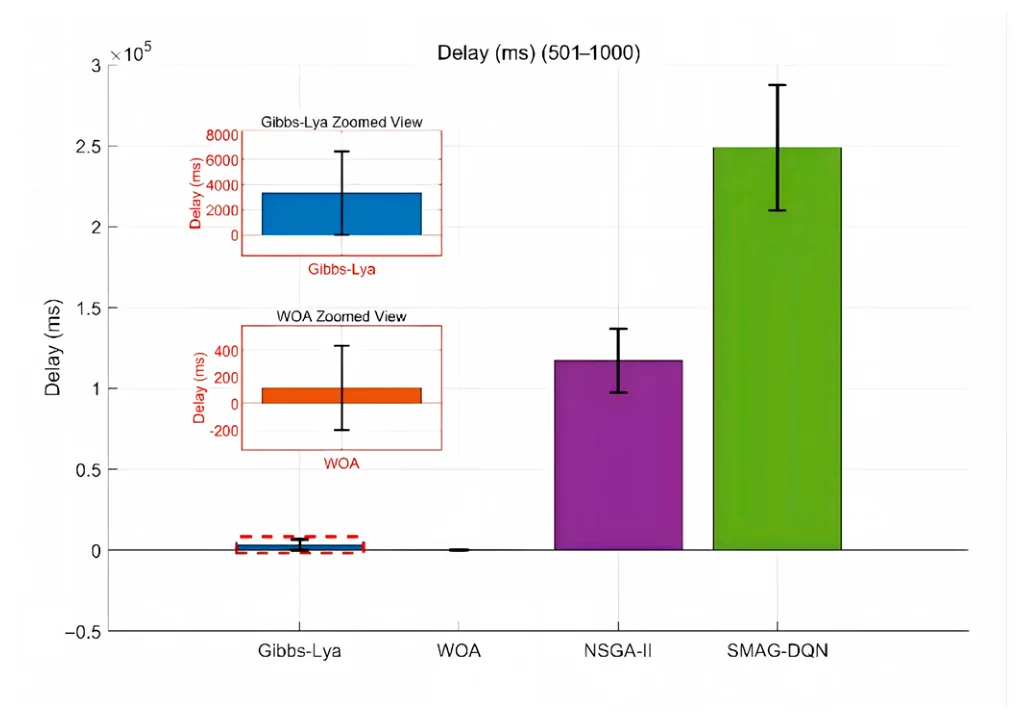
Comparison of Delay.

**Figure 17:**
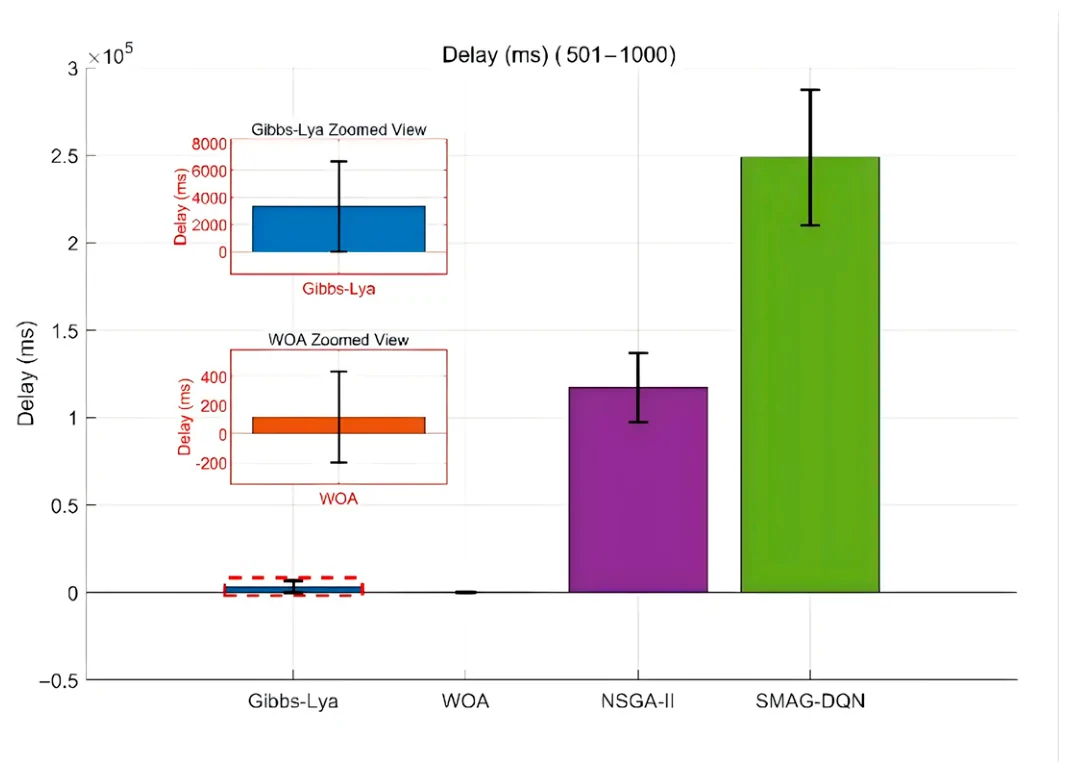
Comparison of Migration energy cost.

**Figure 18:**
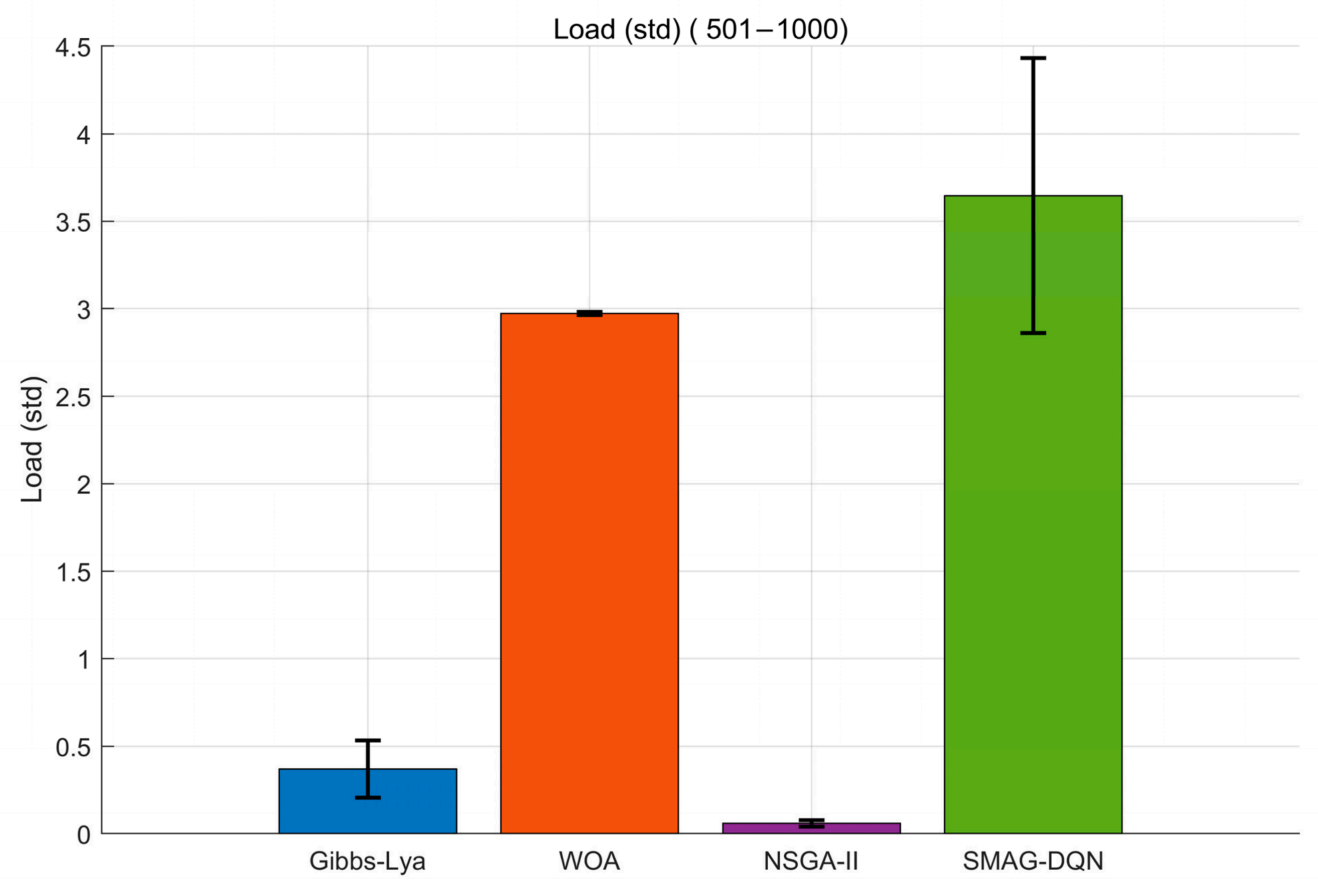
Comparison of Load balancing.

**Figure 19:**
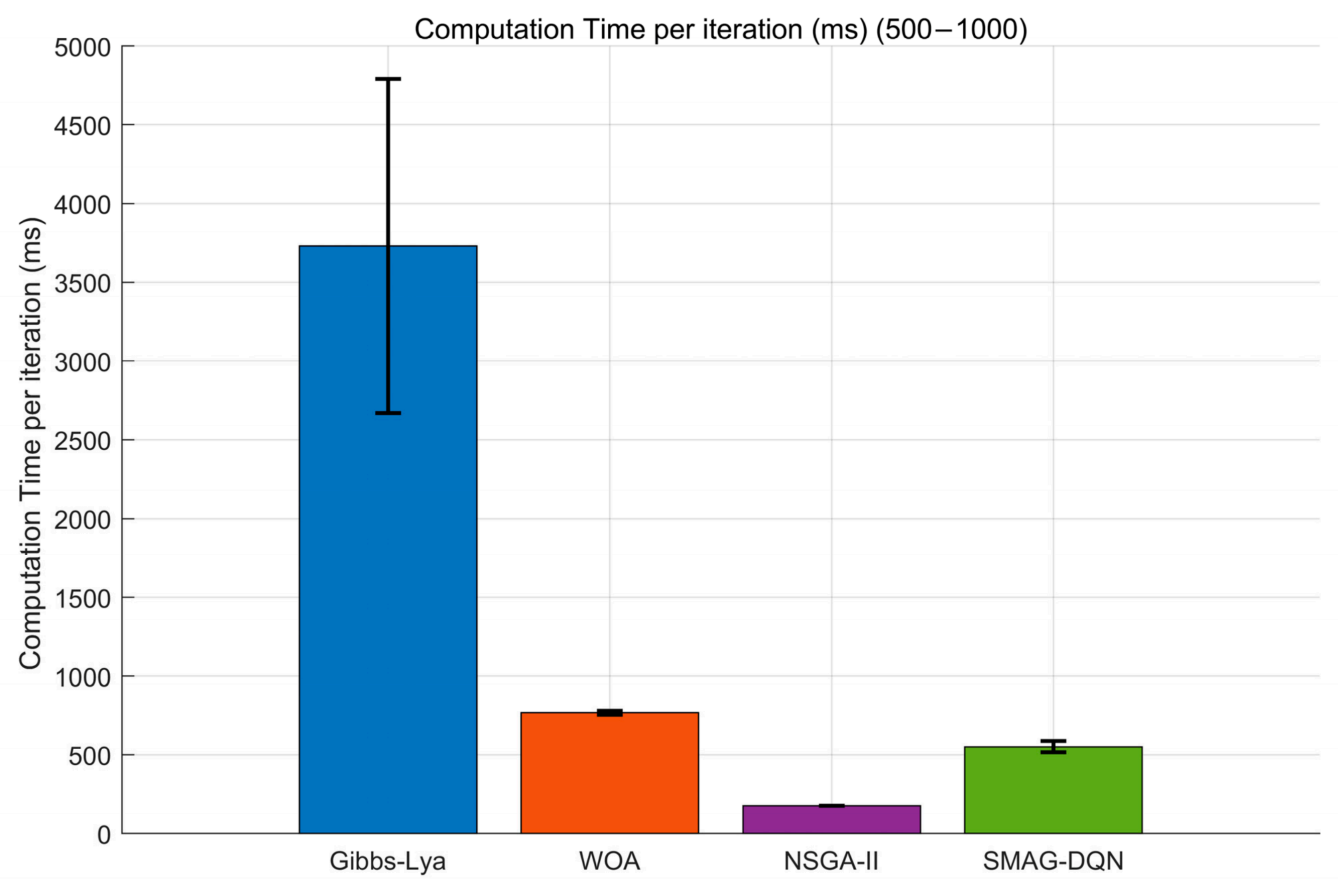
Comparison of per-episode computation time.

As shown in Figure 15, Figure 16, Figure 17, Figure 18 and Figure 19 and summarized in Table 5, the simulation results of the four algorithms reveal a significant positive correlation between load balancing performance and migration overhead.

The WOA achieves the lowest migration delay (118 ms) and migration energy cost (8J) among all compared algorithms, with an intra-vehicle service migration rate of 100% and no inter-vehicle task migration occurring. The training results fully converge to intra-vehicle migration, and the average computation time per iteration is approximately 0.17 s.

The Gibbs-Lyap algorithm demonstrates favorable performance across the four evaluated metrics. Its migration delay (3295 ms) and migration energy cost (194J) rank second only to WOA, while its load balancing rate (0.37) ranks second only to NSGA-II. The intra-vehicle task retention ratio remains stable within the range of 60–70%, with inter-vehicle scheduling performed only for a small number of tasks exhibiting significant load deviations, resulting in a relatively low migration frequency. However, this algorithm suffers from a notable deficiency in computational rapid performance, with an average computation time of 3.73 s per iteration, rendering it unsuitable for time-sensitive dynamic vehicular scenarios.

The NSGA-II algorithm achieves the best load balancing performance (0.06) and the lowest computational complexity (0.18 s per iteration). Nevertheless, its extreme inter-vehicle scheduling strategy—characterized by an intra-vehicle task retention ratio of only approximately 15%, leads to a migration delay as high as 117 s and migration energy cost as high as 512 kJ, exceeding those of the other algorithms by two to four orders of magnitude. This renders the algorithm impractical for engineering deployment in UGV formation scenarios.

This migration-delay spread of more than three orders of magnitude: from 118 ms for the intra-vehicle, WOA strategy to 117 s for NSGA-II. This is directly explained by the hot/cold migration mechanism: WOA performs hot migration by injecting only pending state data over the intra-vehicle Gigabit Ethernet, whereas the extreme inter-vehicle scheduling of NSGA-II forces cold migration of full service images over the 200 kbps wireless link, consistent with the transmission-time analysis in Section 3.

From the perspectives of load balancing rate, migration delay, migration energy cost, same-vehicle migration ratio, and computational rapid performance, a significant positive correlation is observed between load balancing performance and migration overhead: a higher intensity of inter-vehicle scheduling leads to better load balancing, yet incurs higher migration delay and migration energy cost.

#### 5.3.2. Comprehensive Performance Summary

The WOA-based intra-vehicle migration strategy performs service migration with minimal communication energy overhead, enabling rapid recovery of failed services without interrupting the normal functionality of the formation. Consequently, the impact of the resulting load imbalance is strictly confined within the faulty vehicle and does not propagate to other vehicles in the formation. Thus, the overall load balancing pattern established during the static deployment phase is preserved at the formation level.

The SMAG-DQN-based inter-vehicle scheduling strategy offers three key advantages over other algorithms.

1. It achieves a computation time of 0.55 s per iteration, which is approximately 6.8 times faster than Gibbs-Lyap (3.73 s) and only marginally slower than the lightweight NSGA-II. Although Gibbs-Lyap exhibits better performance in other migration metrics, its excessive decision latency renders it unsuitable for time-critical scenarios with random processor failures.
2. Moderate inter-vehicle scheduling strategy: The intra-vehicle migration ratio of SMAG-DQN is approximately 38%, positioned between Gibbs-Lyapunov (64%) and NSGA-II (15%). Compared with WOA, which completely abandons inter-vehicle migration, SMAG-DQN leverages the MAG clustering mechanism to guide selective inter-vehicle migrations, achieving global load balancing improvement across the formation at an acceptable cost of delay and energy consumption. Compared with NSGA-II, which adopts an extreme inter-vehicle scheduling policy, SMAG-DQN reduces migration delay and energy consumption by approximately 2 times and 57 times, respectively, thereby mitigating the risk of communication system collapse.
3. Decision stability: By employing MAG-based batch decision-making, SMAG-DQN compresses the decision chain length from 78 steps to approximately 15–20 steps, significantly suppressing the gradual accumulation of Q-value estimation errors. The coefficient of variation (CV) of migration delay is only 5.7%, substantially lower than the 25.7% observed in the baseline DQN, indicating that the incorporation of clustering prior knowledge effectively reduces the algorithm’s sensitivity to random seeds and yields more stable policy outputs across multiple independent runs.

In summary, for UGV formation fault recovery scenarios characterized by limited onboard computing and communication resources, the WOA-based intra-vehicle migration strategy effectively avoids unnecessary inter-vehicle communication overhead. For severe fault scenarios where the faulty vehicle lacks sufficient residual capacity to accommodate the failed services, the SMAG-DQN-based inter-vehicle load balancing migration strategy provides a feasible solution that simultaneously satisfies rapid decision constraints and acceptable migration overhead, achieving a comprehensive balance among scheduling efficiency, resource utilization, and service quality.

NSGA-II achieves the best load balancing performance and the lowest computational complexity, yet its excessive inter-vehicle scheduling results in migration delay and migration energy cost that far exceed those of the other algorithms. WOA achieves the lowest migration overhead, but completely abandons global load scheduling, leading to extremely low cluster resource utilization and an inability to cope with complex scenarios characterized by dynamic fluctuations in task loads. Gibbs-Lyap performs at a moderate level across all dimensions, yet its significant deficiency in computational rapid performance renders it incapable of meeting the rapid scheduling requirements of dynamic vehicular scenarios. Those three algorithms exhibit a clear single-objective bias.

The proposed SMAG-DQN algorithm achieves a well-balanced optimization across multiple performance dimensions, which better aligns with the engineering requirements of dynamic service migration in UGV formations. Specifically:

1. The computational efficiency of SMAG-DQN is close to that of the lightweight WOA and significantly superior to that of the Gibbs-Lyap algorithm, thus meeting the rapid scheduling requirements of onboard platforms.
2. The migration delay and migration energy cost of SMAG-DQN are only slightly higher than those of WOA, yet are reduced by an order of magnitude compared with the NSGA-II algorithm, effectively ensuring the timeliness of service migration.
3. Although the load balancing performance of SMAG-DQN is inferior to that of the global optimization algorithm, it outperforms WOA in terms of multi-objective optimization imbalance, thereby improving the overall resource utilization of the cluster.

Overall, the SMAG-DQN algorithm achieves a comprehensive optimization of scheduling efficiency, resource utilization, and service quality under the core constraints of limited onboard computational resources and communication energy sensitivity.

## 6. Conclusions

While existing service-migration methods mainly target static or generic edge-computing settings and rarely establish hierarchical adaptive migration mechanisms aligned with fault severity, vehicle residual capacity, and mission phases for UGV formations, this paper addresses this distinct research gap by focusing on fault-induced dynamic migration in cooperative UGV formation scenarios and proposes a fault-severity-aware dual-strategy hierarchical dynamic service migration method.

For future research directions, we intend to perform experimental validation of the proposed strategies on actual physical platforms. It should be noted that the present results demonstrate computational feasibility under simulation conditions only, rather than validated real-time performance on embedded UGV hardware. This will be enabled by the ongoing hardware development of open-architecture UGVs and the physical prototypes to be produced from the unmanned vehicle formation research and development project.

## Figures and Tables

**Table 1:** Notation.

Symbol	Description
*S* = {1, 2, …, *K*}	Set of services to be migrated
*P* = {1, 2, …, *N*}	Set of healthy processors (all available processors)
*CV*(*p*)	UGV to which processor *p* belongs
*CV_fault_*	Faulty vehicle
*S*_local_ = {*p* ∈ *P*|*CV*(*p*) = *CV_fault_*}	Healthy processors on the same UGV as the faulty one
*S*_remote_ = {*p* ∈ *P*|*CV*(*p*) ≠ *CV_fault_*}	Healthy processors on other UGV
*D_state_*_,*s*_	Amount of state data to be migrated for service *s*
*D_image_*_,*s*_	Image size of service *s* to be migrated
*d*_fault,*p*_	Communication distance involved in service migration
*R*_comm_(*d*)	Inter-vehicle communication data rate
*B_app_*	Effective application-layer bandwidth of the Ethernet
*C_p_*	Residual capacity of processor *p*

**Table 2:** Key algorithm parameters.

Training Episodes	1000
Independent runs	10
QN—learning rate	10^−4^
DQN—discount factor γ	0.95
DQN—exploration ε (start/end/decay)	1.0/0.01/0.99
DQN—replay buffer size	50,000
DQN—mini-batch size	128
DQN—target network update interval	5 episodes
DQN—network architecture	64–64–32–18
DQN—optimizer	Adam (β_1_ = 0.9, β_2_ = 0.999)

**Table 3:** Comparison of objective function simulation results.

Objective Function	SMAG-DQN	Baseline DQN
Total reward	−116.11 ± 2.33	−125.84 ± 5.04
Load balancing rate	3.65 ± 0.26	4.59 ± 0.28
Migration delay(ms)	248,621 ± 14,244	110,567 ± 28,448
Migration energy cost (J)	9040 ± 1293	3194 ± 1168

**Table 4:** Comparison of objective CVs.

CV	SMAG-DQN	Baseline DQN
Total reward	2.0%	4.0%
Load balancing rate	7.1%	6.1%
Migration delay(ms)	5.7%	25.7%
Migration energy cost (J)	14.3%	36.6%

**Table 5:** Multi-dimensional simulation comparison of algorithms.

Metric	Gibbs-Lyap	WOA	NSGA-II	SMAG-DQN
Migration delay (ms)	3295 ± 3326	118 ± 314	117,099 ± 19,919	248,621 ± 38,788
Load balancing rate (std)	0.3678 ± 0.1644	2.9731 ± 0.0084	0.0588 ± 0.0193	3.6459 ± 0.7854
Migration energy cost (J)	194 ± 194	8 ± 44	511,584 ± 165,330	9041 ± 3441
Same-vehicle migration ratio (%)	64.4 ± 35.7	100.0 ± 0.2	15.5 ± 7.7	38.0 ± 9.0
Time per-episode (ms)	3731.3092 ± 1060.1269	767.6517 ± 12.2026	177.5210 ± 0.7639	551.4080 ± 36.1240

## Data Availability

The raw data supporting the conclusions of this article will be made available by the authors on request.
